# Glycosaminoglycan-functionalized hydrogels for sustained delivery of tissue inhibitor of metalloproteinase-3 mediating matrix metalloprotease inhibition and extracellular matrix stabilization

**DOI:** 10.1016/j.bioactmat.2026.02.010

**Published:** 2026-02-12

**Authors:** Fabian Junker, Stefan Rupf, Paula Marie Schindler, Cedric Wilden, Mathias Hohl, Gloria Ruiz-Gómez, M. Teresa Pisabarro, Selina Wrublewsky, Caroline Bickelmann, Charlotte Berhorst, Dalia Alansary, Ben Wieland, Markus Bischoff, Poh Soo Lee, Stephanie Moeller, Albrecht Berg, Tobias A. Dancker, Marcel A. Lauterbach, Bergita Ganse, Leticia Prates Roma, Therese Steudter, Wolfgang Metzger, Thomas Tschernig, Emmanuel Ampofo, Matthias W. Laschke, Matthias Hannig, Sandra Rother

**Affiliations:** a Institute of Biophysics, Center of Integrative Physiology and Molecular Medicine (CIPMM), Saarland University, 66421, Homburg, Germany; b Clinic of Operative Dentistry, Saarland University, PharmaScienceHub (PSH), Kirrberger Str. 100, Building 73, 66421, Homburg, Saar, Germany; c Institute for Clinical and Experimental Surgery, Saarland University, PharmaScienceHub (PSH), 66421, Homburg, Germany; d Department of Internal Medicine III - Cardiology, Angiology and Intensive Care Medicine, Saarland University Hospital, Saarland University, Homburg, Germany; e Structural Bioinformatics, BIOTEC Technische Universität Dresden, Tatzberg 47/51, 01307, Dresden, Germany; f Molecular Biophysics, Saarland University, Homburg, Germany; g Institute of Medical Microbiology and Hygiene, Saarland University, 66421, Homburg, Germany; h Institute of Materials Science, Max Bergmann Center of Biomaterials, Technische Universität Dresden, 01069, Dresden, Germany; i Biomaterials Department, INNOVENT e.V., 07745, Jena, Germany; j Molecular Imaging, Center for Integrative Physiology and Molecular Medicine (CIPMM), Saarland University, Kirrberger Str. 100, Building 48, 66421, Homburg, Saarland, Germany; k Werner Siemens Foundation Endowed Chair of Innovative Implant Development (Fracture Healing), Clinics and Institutes of Surgery, Saarland University, 66421, Homburg, Germany; l INM-Leibniz Institute for New Materials, Chemistry Department, Saarland University, 66123, Saarbrucken, Germany; m Department of Trauma, Hand and Reconstructive Surgery, Saarland University, 66421, Homburg, Germany; n Institute of Anatomy and Cell Biology, Saarland University, Homburg, Germany

**Keywords:** Tissue inhibitor of metalloproteinase-3 (TIMP-3), Matrix degradation, Glycosaminoglycans (GAGs), Matrix metalloprotease-9 (MMP-9), Hydrogel

## Abstract

Excessive protease activity and impaired tissue regeneration are hallmarks of many disease states. Elevated matrix metalloproteinase-9 (MMP-9) plays a key role in adverse tissue remodeling by excessively degrading extracellular matrix (ECM) components and growth factors. Tissue inhibitor of metalloproteinase-3 (TIMP-3) regulates ECM turnover, and its bioavailability is influenced by glycosaminoglycans (GAGs). This study aimed to develop a methacrylated gelatin (GelMA)-based hydrogel functionalized with acrylated sulfated hyaluronan (sHA_c_) as a TIMP-3 delivery system to decrease ECM degradation under pathophysiological conditions. sHA_c_ incorporation enhanced hydrogel stiffness, reduced degradation rates and yielded sustained TIMP-3 release for up to 28 days. Molecular modeling and surface plasmon resonance demonstrated preferential binding of TIMP-3 to sHA_c_ over hyaluronan methacrylates, together providing a molecular rationale for the reduced and sustained release of TIMP-3 from sHA_c_-containing hydrogels. Angiogenesis-related functional assays, supported by molecular modeling studies, indicate that sHA_c_ modulates the anti-angiogenic activity of TIMP-3 by altering vascular endothelial growth factor receptor-associated signaling, while preserving metalloproteinase inhibition. Released TIMP-3 from GelMA/sHA_c_ hydrogels retained bioactivity, effectively inhibiting MMP-9 activity and mitigating ECM degradation in-vitro and in human ex-vivo models. In a murine subcutaneous implantation model, sHA_c_-functionalized TIMP-3-loaded hydrogels were associated with reduced inflammatory cell presence and altered vascular- and matrix-related tissue signatures compared with GelMA controls. These findings underscore the potential of sHA_c_-functionalized GelMA hydrogels as biomaterials for therapeutics delivery, offering controlled TIMP-3 release and sustained bioactivity to promote ECM stability and on-demand MMP inhibition. This system represents a promising strategy for addressing the challenges of excessive MMP activity.

## Highlights

• sHA_c_ functionalization controls TIMP-3 binding and sustained release.
• sHA_c_ modulates TIMP-3 anti-angiogenic activity via VEGFR-associated signaling.
• GelMA/sHA_c_ hydrogels deliver bioactive TIMP-3 in-vitro and ex-vivo.
• GelMA/sHA_c_ implants show reduced inflammation in a murine subcutaneous model.
• The platform attenuates excessive protease-driven ECM degradation ex-vivo.

## Graphical abstract

**Figure:**
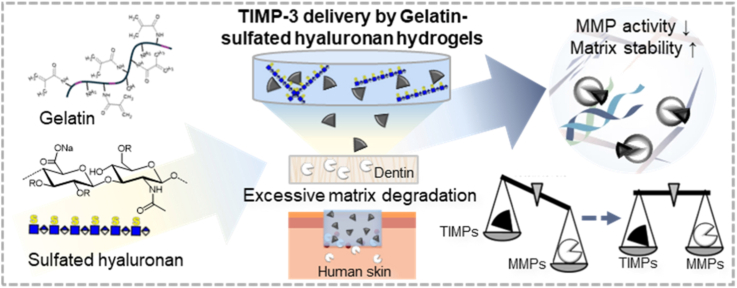

## 1 Introduction

Excessive extracellular matrix (ECM) degradation driven by matrix metalloproteases (MMPs) plays a central role in pathophysiological conditions, such as chronic diabetic wounds, cardiovascular diseases and dental restoration failure. In these settings, protease-rich microenvironments drive sustained tissue damage and contribute to a significant global healthcare burden [1,2]. The increasing prevalence of these diseases is closely linked to an aging population and the rising incidence of diabetes and obesity [[3], [4], [5], [6]]. The proteolytic activity of MMPs is required for fundamental cell functions like cell migration and morphogenesis and is under strict control especially by tissue inhibitors of metalloproteases (TIMPs) [7]. However, under pathophysiological conditions, elevated levels of proteases, particularly MMP-9, drive excessive degradation of ECM components and growth factors, leading to maladaptive alterations in tissue architecture [8,9]. Even though MMP inhibition is a promising treatment strategy, systemic administration of protease inhibitor drugs holds the risk of several off-target effects limiting the clinical translation [10].

Thus, there is a high demand for “smart” biomaterials offering a controlled release of bioactive molecules to avoid over-dosing of drugs or recombinant proteins or quick inactivation of bioactive molecules due to degradation [[11], [12], [13], [14]]. Bio-inspired biomaterials that mitigate extensive ECM degradation have garnered significant interest for addressing the challenges of excessive MMP activity [15,16]. One promising approach is the incorporation of protease inhibitors into biopolymer-based hydrogels [[17], [18], [19]]. In particular, methacrylated gelatin (GelMA) hydrogels as water-swollen polymer networks based on processed collagen type I have emerged as versatile materials due to their biocompatibility, biodegradability, tunable mechanical properties, temperature-sensitive shape adaptation and ease of functionalization [[20], [21], [22]]. However, enhancing their bioactivity and ability to protect the ECM from degradation remains a critical area of research.

A promising approach to more accurately replicate the native ECM composition from a physicochemical point of view involves modifying GelMA hydrogels with biomimetic components like glycosaminoglycans (GAGs), such as hyaluronan (HA), chondroitin sulfate (CS), or heparin (Hep) [[23], [24], [25]]. Upon binding, GAGs can modulate protein activity through various mechanisms, including protection from degradation, induction of conformational changes that activate protein function, or formation of complexes with cellular receptors. GAG-protein interactions are predominantly electrostatic, involving negatively charged carboxyl and sulfate groups of the polysaccharides and clusters of basic amino acids on the protein surface. As a result, the binding properties of GAGs are closely associated with their sulfate density and distribution [26,27]. A notable limitation of utilizing natural, predominantly animal-derived GAGs like Hep lies in their inherent batch-to-batch variability. In contrast, biosynthetically produced GAG derivatives provide superior consistency and well-defined, tunable properties [28,29]. Biotechnologically produced HA can serve as building block to synthesize chemically modified glycans from sustainable sources that mimic the properties of naturally occurring sulfated GAGs such as heparan sulfate [30]. In particular, sulfated hyaluronan (sHA) was reported to possess immunomodulatory properties and to sequester proteins through charge interactions [[31], [32], [33], [34]].

Within the native ECM, TIMP-3 is a potent natural inhibitor of all MMPs and a major regulator of ECM turnover, uniquely binding to GAGs to provide localized and sustained inhibition of protease activity [35,36]. Native and chemically modified GAGs have been demonstrated to enhance TIMP-3 stability and activity [37,38]. Furthermore, TIMP-3 has been demonstrated to suppress vascular endothelial growth factor-A (VEGF-A)-induced expression of MMP-2 and MMP-9, as well as to regulate the release of cell membrane-bound tumor necrosis factor-α (TNF-α) [39,40]. Thus, increasing the extracellular TIMP-3 levels with biomaterials is a promising strategy to regulate excessive protease activity that contributes to pathological ECM degradation.

Building on these findings, this study aimed to develop bioactive GelMA-based hydrogels functionalized with sHA as tunable TIMP-3 release systems to reduce excessive MMP activity. Incorporating sHA acrylates (sHA_c_) into the GelMA hydrogel improved the ECM-mimicking characteristics of the gels, which are crucial for creating a biomimetic environment conducive to the release and activity of bioactive proteins. To evaluate the therapeutic potential of this system, an ex-vivo human dentin model and an ex-vivo human skin model were established. Dentin, the collagen-rich connective tissue beneath tooth enamel and cementum, exhibits inherent proteolytic activity when demineralized, closely resembling ECM degradation observed under pathophysiological conditions [2,41]. Moreover, skin biopsies from human body donors with defined induced protease-rich, pro-inflammatory wounds enable direct insight into human tissue responses relevant for translational applications.

Thus, both human models provide a controlled and ethical alternative to in-vivo animal models in accordance with the 3R principle (replace, reduce, refine). By combining sHA_c_ with TIMP-3 within the GelMA scaffold, we designed a hydrogel platform that not only serves as a reservoir for the controlled release of bioactive TIMP-3 but also actively mitigates ECM degradation in-vitro and ex-vivo. Importantly, our findings highlight the potential of bioactive GAG-functionalized hydrogels as advanced biomaterials for on-demand sustained MMP inhibition addressing the unmet need for innovative therapeutic approaches to preserve and restore ECM integrity in various biomedical applications.

## 2 Materials and methods

Lithium phenyl-2,4,6-trimethylbenzoylphosphinate (LAP), GelMA with a degree of substitution of 60 % and a gel strength of 170-195 g Bloom and HA methacrylate (HA_c_) with a degree of substitution of 20 - 50 % and a molecular weight of 20-30 kDa, and Hep from porcine intestinal mucosa were purchased from Merck (Darmstadt, Germany). CS (porcine trachea; mixture of 70 % chondroitin-4-sulfate and 30 % chondroitin-6-sulfate) was purchased from Kraeber (Ellerbek, Germany). Human recombinant TIMP-3 and human recombinant VEGF-A were obtained from Bio-Techne (Wiesbaden, Germany). All chemicals were obtained from Merck (Darmstadt, Germany), unless specified otherwise.

### 2.1 Preparation of sHA_c_

**Fig. 1:**
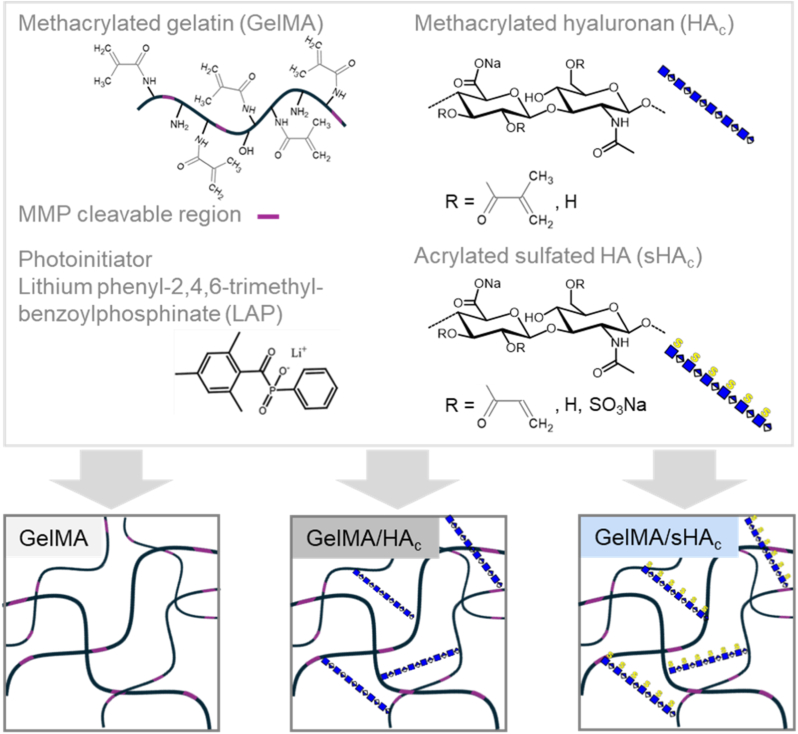
Schematic illustrating the hydrogel compositions used in this study. Methacrylated gelatin (GelMA) served as hydrogel precursor naturally containing MMP-degradable peptide sequences. GelMA exposure to UV light in the presence of the photoinitiator LAP initiated polymerization. To prepare GelMA functionalized with HA or sHA, (meth)acrylated HA_c_ or sHA_c_ derivatives were added before photopolymerization. Polymer network formation was due to crosslinking of the (meth)acrylate side groups of GelMA and HA_c_/sHA_c_.

The sHA_c_ were synthesized from sHA derivatives, featuring sulfate residues at the C6 position, and characterized as reported previously [28]. In brief, sHA was used as substrate for reaction with acryloyl chloride under phase transfer conditions to produce the respective acrylated derivative. The degree of acrylation was estimated from the ^1^H NMR spectra, while the degree of sulfation was calculated based on the sulfur content determined using an elemental analyzer. Molecular weight was analyzed by gel permeation chromatography with refractive index- and laser light scattering detection. Crosslinkable sHA_c_ had a molecular weight of 16 kDa, a degree of sulfate substitution of 1.2 and a degree of acrylate substitution of 25 %. The structure of sHA_c_ is depicted in Fig. 1.

### 2.2 Hydrogel fabrication

The biomaterials were fabricated by combining 55 μL of 10 % (w/w) GelMA, preheated to 37 °C, with 3 μL of phosphate-buffered saline (PBS, pH 7.3, calcium- and magnesium-free; Gibco, Thermo Fisher Scientific, Dreieich, Germany) and 4 μL of 10 mg/mL LAP. The mixture was subjected to UV cross-linking at 365 nm (4 mW/cm^2^) for 60 s, followed by freeze-drying. For the preparation of GAG-containing biomaterials, 3 μL of HA_c_ (20 % w/w) or sHA_c_ (20 % w/w) dissolved in PBS were added to the GelMA/LAP mixture instead of the PBS solution before photopolymerization. The final composition of the prepared hydrogels is summarized in Table 1.

**Table 1:** Hydrogel compositions.

Component	GelMA	GelMA/HA_c_	GelMA/sHA_c_
GelMA	100 %	90.5 %	90.5 %
HA_c_	-	9.5 %	-
sHA_c_	-	-	9.5 %

### 2.3 Biomaterial characterization

The hydrogel stability over time was studied at 37 °C in 500 μL PBS. A defined volume was collected and replaced by fresh PBS after certain time points. The amount of released GelMA was quantified using the BCA assay with GelMA as standard for calibration. The release of HA_c_ was evaluated with the hexosamine assay and the amounts of sHA_c_ in solution were quantified via the dimethylmethylene blue (DMMB) assay) as described previously [42].

For hydrogel swelling, siliconized 96-well plates were employed as molds to fabricate hydrogels for subsequent measurements. The diameter and height of each hydrogel were measured using a caliper, and their weights were recorded. After initial characterization, each lyophilized hydrogel was rehydrated in 1000 μL PBS and incubated at 37 °C. Measurements were repeated after 1 h, 24 h, and 96 h of incubation. Hydrogel swelling was quantified using the swelling ratio formula described previously [43]. Each hydrogel type was tested with a sample size of n = 3.

For scanning electron microscopy (SEM) imaging to analyze the microscopic surface morphology of hydrogels, lyophilized GelMA, GelMA/HA_c_, and GelMA/sHA_c_ hydrogels were used. The samples were mounted onto conductive carbon adhesive tabs and sputter-coated with carbon (SCD 030, Balzers Union, Balzers, Liechtenstein). Imaging was performed under high vacuum conditions with an acceleration voltage of 5 kV in secondary electron mode using a FEI XL 30 ESEM FEG scanning electron microscope (FEI, Hillsboro, USA). The pore size distribution was analyzed from SEM micrographs with ImageJ, and the results were expressed as percent frequency.

### 2.4 Atomic force microscopy (AFM)

AFM surface images of hydrogels were acquired in PBS (pH = 7.4) with a Nanowizard 4 (Bruker Nano GmbH, Berlin, Germany) and SNL10-D cantilevers (Bruker-Nano, Santa Barbara, USA) used in quantitative imaging (QI™) mode. Before each measurement, cantilevers were calibrated using the Sader method [44]. An area of 3 × 3 μm (128 pixels by 128 pixels) was scanned. While scanning the hydrogels, the setpoint was set to 5 nN, the Z-length varied between 1500 nm and 2000 nm, and the Z-speed varied between 20 μm/s and 100 μm/s according to the softness of the imaged hydrogel. AFM images were processed using the JPK SPM Data Processing software, Version 8.1.70.

### 2.5 Mechanical testing

Freeze-dried cylindrical samples from 240 μL polymer mixture were rehydrated in 1 mL PBS at room temperature for 6 h prior to mechanical testing. The E-modulus of each hydrogel was determined using the MicroTester (CellScale Biomaterials Testing, Waterloo, Canada). During testing, the samples were submerged in a water bath containing PBS and all measurements were conducted at room temperature. A cantilever, with a microbeam diameter of 1.016 mm and a 5 × 5 mm square plate, was used to achieve a deflection ratio between 0.55 and 0.70. For each sample, Z-compression with a 30 % amplitude was performed in ramp mode for 120 s. A stress-strain curve was generated for each sample based on the measured force, and the slope up to 15 % strain was used to calculate the E-modulus. Each variant was tested with a sample size of n = 4.

### 2.6 Hydrogel mesh size calculation

The network mesh size (ξ) of rehydrated GelMA hydrogels was estimated from equilibrium swelling and unconfined compressive mechanical data using classical rubber elasticity theory and Gaussian chain statistics, following established approaches [[45], [46], [47]]. After 96 h of swelling in PBS, the equilibrium mass swelling ratio was calculated as Q_m_ = W_s_/W_d_, where W_s_ and W_d_ are the swollen and dry masses, respectively. The polymer volume fraction in the swollen state was obtained by applying a density correction according to Φ_2_,_s_ = 1/[1 + (ρ_p_/ρ_s_)(Q_m_ – 1)], with polymer density ρ_p_ = 1.35 g/cm^3^ and solvent density ρ_s_ = 1.00 g/cm^3^. Unconfined compression tests at room temperature provided the Young's modulus E, determined from the initial slope of the stress-strain curve up to approximately 15 % strain. Assuming near-incompressibility of swollen hydrogels (Poisson's ratio ≈ 0.5), the shear modulus was derived as G = E/3. The average molecular weight between crosslinks was then estimated from rubber elasticity using M_c_ = (ρ_p_ × Φ_2_,_s_ × R × T)/G, where R = 8.314 J mol^−1^ K^−1^ and T = 295 K. Finally, the mesh size was calculated according to Gaussian chain statistics as ξ = (2 × C_∞_)^1/2^ × l × (M_c_/M_r_)^1/2^ × Φ_2_,_s_^−1/3^, with the characteristic ratio C_∞_ = 9, average bond length l = 0.15 nm, and average molecular weight of a repeating gelatin unit M_r_ = 91 g/mol. For each specimen, ξ was calculated by pairing its corresponding E and Q_m_ values, and results are reported as mean ± standard deviation. The internal void fraction of rehydrated hydrogels was determined from equilibrium swelling as *ε* = 1 – Φ_2_,_s_.

### 2.7 Enzymatic hydrogel degradation *in-vitro*

Hydrogels from 100 μL polymer mix were incubated with 357 U/mL *Clostridium histolyticum* collagenase (CHC) and 500 U/ml hyaluronidase in 50 mM Tris, 10 mM CaCl_2_, 150 mM NaCl, and 0.05% Brij-35, pH 7.5 for 1 h and 24 h, respectively. Residual GelMA was quantified using a BCA assay, with the corresponding enzyme degradation solution serving as the blank for background correction. The release of sHA_c_ was analyzed using the DMMB assay, and the total amount of liberated HA_c_ was quantified by turbidity measurements at 600 nm after sample incubation with cetyltrimethylammonium bromide (CTAB) as described in Ref. [48]. Supernatants collected after enzymatic degradation were further analyzed by agarose gel electrophoresis following ethanol precipitation of GAGs to remove impurities. Electrophoresis was carried out on a 1 % agarose gel in Tris-acetate-EDTA (TAE) buffer for 3 h at 100 V, preceded by a 6 h pre-run at 80 V, as previously described [49], to ensure sufficient separation of GAGs. HA size standards (Amsbio, Cologne, Germany), together with HA_c_, sHA_c_, and high- and low-molecular-weight HA (molecular weights 1433 kDa and 35 kDa), were used for band identification after staining with Stains-All. Gels were imaged with an Epson Perfection V850 Pro scanner. Each hydrogel type was tested with a sample size of n = 4 for each time point.

### 2.8 Oscillatory shear rheological analysis

The rheological behavior and crosslinking kinetics of the hydrogels were characterized using a rotational rheometer (DHR3, TA Instruments, USA) equipped with a 12 mm parallel plate geometry as described in Ref. [50] with some slight modifications. In brief, the lower plate was transparent and connected to a UV/Vis light source (Omnicure Series 1500) with a 365 nm filter, allowing in situ photopolymerization at room temperature using TRIOS software. Hydrogel precursor solutions of GelMA, GelMA/HA_c_ and GelMA/sHA_c_ were prepared as described above. A 38 μL aliquot of each formulation was dispensed onto the lower plate using a micropipette, and the upper plate was lowered to a fixed gap of 300 μm. To minimize evaporation, the sample was surrounded with silicone oil and covered with a metal enclosure to prevent external disturbances during measurement. To identify the linear viscoelastic region (LVR), strain sweeps (0.1 to 100 % strain at 1 Hz) and frequency sweeps (0.01 to 100 Hz at 0.1 % strain) were conducted. Time sweep experiments were then performed under the established LVR conditions, with an applied strain of 0.1 % and frequency of 1 Hz. Storage (G′) and loss (G″) moduli were recorded in real time for 10 min. After 60 s, the samples were irradiated for 60 s (365 nm, 10 mW/cm^2^) to induce crosslinking. The gelation point (t_gel_) defined as the time after start of the exposure at which G′ equals G″ was used to compare the crosslinking kinetics of different hydrogel compositions. The final storage modulus of the gels was assessed by measuring G′ after 10 min. Each hydrogel type was tested with a sample size of n = 3.

### 2.9 Surface plasmon resonance (SPR) analysis

Binding analysis was performed using a Biacore™ T200 (Cytiva). TIMP-3 was immobilized onto Series S CM5 sensor chips via amine coupling at 25 °C following the manufacturer's instructions. On average, 4295 RU of TIMP-3 were immobilized. An activated and subsequently deactivated flow cell was used as a reference surface without immobilized protein. The running buffer consisted of 50 mM Tris, 10 mM CaCl_2_, 150 mM NaCl, and 0.05 % Brij-35, pH 7.5, and was used both for sample dilutions and during the interaction studies. Samples were injected for 240 s at a flow rate of 30 μL/min. GAGs were injected at concentrations of 100, 200, and 600 μM relative to their disaccharide units (D.U.), and binding levels were recorded 10 s before the end of each injection. Native HA, CS, and Hep were included as reference controls, in addition to sulfated HA with (sHA_c_) or without crosslinkable acrylate groups (sHA) and methacrylated HA (HA_c_). The dissociation phase was monitored in running buffer for 10 min at a flow rate of 30 μL/min. Following each sample injection, the chip surface was regenerated with 5 M NaCl for 60 s, and the baseline was allowed to stabilize for 1000 s prior to the next injection. Data were processed using the Biacore T200 Evaluation Software. Specific binding sensorgrams were obtained by double referencing, subtracting both the response of the reference flow cell and the buffer-only injections.

### 2.10 TIMP-3 binding and release studies

Hydrogels with a diameter of 1.5 mm were incubated with 50 ng TIMP-3 in simulated body fluid (SBF) supplemented with 1 % bovine serum albumin (BSA) at 37 °C overnight. SBF was prepared according to the established Kokubo protocol, using the standard ionic concentrations (mM): Na^+^ 142, K^+^ 5, Mg^2+^ 1.5, Ca^2+^ 2.5, Cl^−^ 148, HCO_3_^−^ 4.2, HPO_4_^2−^ 1.0, SO_4_^2−^ 0.5 adjusted to pH 7.40 [51], which reproduces the inorganic ion concentrations of human plasma. After defined timepoints, the supernatants were collected and stored at −20 °C until further use and replaced by PBS. The amounts of TIMP-3 in the supernatants were quantified by sandwich ELISA (Bio-Techne, Wiesbaden, Germany) according to the manufacturer's protocol. Each gel type was tested with a sample size of n = 3 for each time point.

### 2.11 Immunofluorescence staining of TIMP-3 within the hydrogels

Hydrogels were loaded with TIMP-3 as described above and fixed with 4 % paraformaldehyde (PFA) for 30 min after either 1 h or 672 h of release in SBF. Following three washes with PBS, samples were blocked for 10 min in 1 % BSA and 0.05 % Tween-20 in PBS. TIMP-3 was detected using a human TIMP-3 monoclonal antibody (MAB973, Bio-Techne, Germany). Hydrogels were incubated with the primary antibody for 60 min at room temperature, washed three times with PBS, blocked again for 10 min, and then incubated with the ATTO 647N-conjugated secondary antibody for 60 min. After two final PBS washes, hydrogels were stored in PBS and imaged immediately. Depth images of the hydrogel were acquired using a confocal microscope previously described by Staudt et al. [52]. A 60x water immersion objective (NA 1.2, UPLXAPO60XW, Olympus Germany, Hamburg, Germany) was used to match the refractive index of the hydrogels as closely as possible. The fluorophore ATTO 647N was excited using a 640 nm laser. The detection window was 650 nm to 720 nm. Depth profiles were acquired scanning approximately 200 μm into a hydrogel (z-axis) along a single line (x-axis) with a pixel size of 80 x 80 nm. ImageJ was used to visualize the signal intensity distribution along the z-axis, averaging the fluorescence signal across all horizontal pixels.

### 2.12 Molecular modeling

For modeling purposes, and based on previous work [53], GAG derivatives consisting of six saccharide units (i.e. hexasaccharides) were considered as representative of polymeric forms. Given the experimental ratio of methacrylation in HA_c_ and that of sulfation and acrylation in sHA_c_ (see section “Preparation of sHA_c_), the structures of the following GAG molecules were prepared using AMBER19 [54] and MOE [55] as previously described [33]: HA_c_ methacrylated at position C6 of each disaccharide unit (*i.e.* HA_6 MC), sHA_c_ sulfated at position C6 at each disaccharide unit and acrylated either at position C2′ or C3’ in only one disaccharide unit of the corresponding hexasaccharide (*i.e.* HA6_2AC1 and HA6_3AC1).

#### 2.12.1 Molecular docking of GAGs to TIMP-3

GlycoTorch Vina [56] was used to dock each GAG derivative to a previously reported 3D model of TIMP-3 [57]. A box with dimensions 63 x 63 × 63 Å was used for the protein, which was treated rigidly. The GAG molecules were considered flexible. The exhaustiveness of the search algorithm and number of decoys were set up to 100. The top 50 docking solutions were clustered with DBSCAN as previously described [58]. Three binding poses from each cluster were selected as representatives for further refinement.

#### 2.12.2 Molecular docking of VEGF receptor-2 (VEGFR-2) to TIMP-3

The protein-protein docking protocol from MOE [55] was used for predicting binding of TIMP-3 to a previously reported 3D model of VEGFR-2 [59]. Both proteins were considered rigid. The hydrophobic patch potential and the rigid body method with default parameters for pose refinement were used. The top 10 solutions were analyzed in the context of the structure of the ADAM/TIMP-3 and the MD-based refined GAG/TIMP-3 (see below) complexes.

#### 2.12.3 Molecular dynamics (MD) simulations

The selected representative GAG/TIMP-3 complex structures were energetically refined by MD simulations in AMBER19 [54]. Charges were taken from the GLYCAM 06-j force field [60] and from literature for sulfate groups [61]. RESP atomic charges [62,63] were derived at the HF/6-31G(d) calculation level for the methacrylate and acrylate fragments using Gaussian09 [64]. Parameters for the GAG part were taken from the GLYCAM-06j force field [60] and from the ff14SB force field [54] for the proteins. Missing parameters for the methacrylate and acrylate groups were taken from the General Amber Force Field (GAFF2) [65]. Each GAG/protein complex was solvated in a truncated octahedral box of TIP3P water molecules and neutralized with Cl^−^ counterions. MD simulations were preceded by two energy minimization steps: *i*) only the solvent and ions were relaxed with position restraints for the solute (500 kcal/mol·Å^2^) using 1000 steps of steepest descendent followed by 500 steps of conjugate gradient minimization; *ii*) the entire system was minimized without restraints applying 3000 cycles of steepest descendent and 3000 steps of conjugate gradient equilibration. Then, the system was heated up from 200 to 300 K in 20 ps with weak positional restraints (10 kcal/mol·Å^2^). Langevin temperature coupling with a collision frequency γ = 1 ps^−1^ was used at this step. The system was equilibrated under constant pressure of 1 atm using periodic boundary conditions (NPT) at 300 K for 500 ps. A total of 40 ns MD simulation was carried out at 300 K NPT conditions for each complex. The SHAKE algorithm was used to constrain all bonds involving hydrogens with a time step of 2 fs. A cutoff of 8 Å was set to treat non-bonded interactions, and the Particle Mesh Ewald (PME) method was applied to treat long-range electrostatic interactions. MD trajectories were recorded every 10 ps. The GAG pyranose rings were harmonically restrained. Trajectories were visualized with VMD [66]. Energy decomposition per residue as well as free energy of binding post-processing analysis of 200 frames distributed in the last 20 ns of MD simulations were performed in implicit solvent using the MM-GBSA method [67,68] as implemented in AMBER. Data analysis was carried out with OriginLab [69]. Figures were created with PyMOL [70].

### 2.13 MMP-9 activity assay

Hydrogels were preloaded with 200 ng TIMP-3 in MMP buffer (50 mM HEPES, 10 mM CaCl_2_, 0.05 % Brij-35, pH 7.5) overnight at 37 °C. Afterwards, the supernatants were collected and replaced by fresh MMP buffer. The MMP-9 activity was determined using the MMP-9 fluorometric drug discovery kit from Enzo Life Sciences (Lörrach, Germany). Each gel type was tested with a sample size of n = 3 for each time point.

The bioactive fraction of TIMP-3 relative to the total amount of TIMP-3 released from the hydrogels was expressed as a fold change compared to GelMA hydrogels without GAGs. This was determined using a four-parameter logistic regression model (log[inhibitor] vs. response curve) fitted in GraphPad Prism 10 (GraphPad Software, San Diego, USA), with defined TIMP-3 concentrations utilized to assess MMP-9 inhibition. The remaining bioactivity of hydrogel-released TIMP-3 defined as inhibitory potentials against MMP-9 was divided by the total amount of hydrogel-released TIMP-3 at each time point. This normalization accounted for variations in TIMP-3 release across conditions. The bioactivity of TIMP-3 after 1 h of incubation was designated as 100 %.

### 2.14 Fibroblast-based protease activity assay

Normal human dermal fibroblasts (NHDFs) (PromoCell GmbH, Heidelberg, Germany), were cultured in Dulbecco's modified eagle medium (DMEM) with 10 % fetal calf serum (FCS) and 1 % streptomycin and penicillin at 37 °C at 80 % confluency in 175 cm^2^ flasks. To stimulate protease secretion, cells were treated with 20 ng/mL human recombinant TNF-α (Bio-Techne, Wiesbaden, Germany) for 48 h. The supernatants were collected and used to determine the protease activity in the presence or absence of soluble HA_c_ and sHA_c_ with or without additional TIMP-3 or hydrogel extracts as described in section “cytotoxicity of hydrogel extracts” using the EnzChek Gelatinase/Collagenase Assay Kit (Fisher Scientific, Schwerte, Germany) according to the manufacturer's protocol.

### 2.15 ECM degradation assay

Collagen-based ECMs were prepared in 48-well plates by coating each well with 200 μL 1 mg/mL collagen type I (Corning, Amsterdam, The Netherlands). The neutralized collagen mixture was incubated at 37 °C for 2 h for in-vitro fibrillogenesis as described previously [49]. Dried collagen-based ECMs were incubated with 100 μL 30 U/mL CHC (Merck, Darmstadt, Germany) in PBS and 150 μL test solution from TIMP-3-loaded biomaterials for 20 or 60 min at 37 °C. The enzymatic ECM degradation was stopped by adding 100 μL/well 0.2 M EDTA solution. ECM degradation was assessed by staining the remaining collagen with Sirius red (1 mg/mL Sirius red in saturated picric acid) for 15 min at room temperature followed by removal of non-bound dye with 0.01 M HCl. The matrix-bound dye was eluted using 0.1 M NaOH and the absorbance of obtained solutions was measured at 540 nm to assess the relative amount of remaining matrix [71]. Each variant was tested with a sample size of n = 3 for the individual time points.

### 2.16 Tube formation assay

HUVEC (human umbilical vein endothelial cells) were seeded in a 96-well plate (1.5 × 10^4^ cells per well), which contained 50 μL growth-factor-reduced Matrigel per well. The cells were exposed to TIMP-3 (10 ng/mL dissolved in sterile water), HA_c_ (300 μg/mL dissolved in sterile water), sHA_c_ (300 μg/mL dissolved in sterile water), combinations of TIMP-3 and each of the GAGs or vehicle. Phase-contrast light images were taken after 6 h. Tube formation was quantified by means of measuring the number of tube meshes using FIJI software (U.S. NIH).

### 2.17 Proteome profiler array

For the preparation of cell lysates, HUVEC samples were lysed for 30 min at 4 °C in radioimmunoprecipitation assay buffer (Thermo Scientific, Germany) containing 0.5 mM phenylmethylsulfonyl fluoride (PMSF) and a protease/phosphatase inhibitor cocktail (Cell Signaling Technology, USA). Total protein concentration was quantified by means of a Bradford assay using a NanoPhotometer NP80 (Implen, Germany). Proteome profiling was performed by using the proteome profiler human angiogenesis kit ARY007 (R&D systems, USA). Proteome array membranes were blocked by incubation with array buffer 7 for 1 h on a rocking platform. While blocking, 200 μg of each sample were adjusted to a final volume of 1.5 mL by using the array buffers 4 and 5. Samples were incubated for 1 h after adding 15 μL detection antibody cocktail to each of them. Subsequently, array buffer 7 was aspirated and sample/antibody mixtures were added to the respective array membrane which were incubated overnight at 4 °C on a rocking platform. The next day, array membranes were washed with 1 x wash buffer for 10 min. This step was repeated for a total of three times. After washing, array membranes were incubated with streptavidin-HRP for 30 min. Following to that, array membranes were washed again for three times. Finally, proteins were visualized by chemiluminescence in a Chemocam device (Intas, Göttingen, Germany).

### 2.18 Isolation of microvascular fragments (MVF)

For the isolation of MVF, male and female C57/BL6J mice with a body weight of about 30 g were anesthetized by an i.p. injection of ketamine (100 mg/kg body weight) and xylazine (12 mg/kg body weight). The epididymal fat pads were harvested, washed and mechanically dissected before the tissue was enzymatically digested by collagenase IAS (from clostridium histolyticum, Sigma-Aldrich, USA) for 9 min. After their isolation, MVF were transferred in DMEM (4.5 g/L Glucose), (10 % FCS, 100 U/mL penicillin and 0.1 mg/mL streptomycin).

### 2.19 Generation of MVF spheroids and sprouting assay

After isolation, MVF spheroids were generated by means of the liquid overlay technique in a 96-well plate covered with 1 % agarose. After 5 days, the MVF spheroids were harvested, and the influence of TIMP-3, HA_c_ and sHA_c_ on their angiogenic activity was determined by a sprouting assay. For this purpose, the MVF spheroids were embedded into a collagen solution which was stabilized by incubation at 37 °C. After 45 min, DMEM supplemented with 10 % FCS, 100 U/mL penicillin, 0.1 mg/mL streptomycin and the respective treatment (10 ng/mL TIMP-3, 300 μg/mL HA_c_, 300 μg/mL sHA_c_ and combinations of TIMP-3 with each of the GAGs) was added for 72 h at 37 °C and 5 % CO_2_. The sprouted MVF spheroids were visualized by a Keyence microscope BZ-X810 (KEYENCE, Frankfurt am Main, Germany). The sprouting area was assessed by means of the Fiji software (NIH). Data are given in % of the initial spheroid area (day 0).

### 2.20 VEGF-A signaling bioassay

A VEGF bioassay using genetically engineered KDR/NFAT-RE HEK293 cells was performed according to the manufacturer's protocol (Promega, Mannheim, Germany). Biomaterials were loaded with 16.7 ng/μL TIMP-3 and incubated in DMEM without supplements overnight. Afterwards, the supernatants were collected and replaced by fresh media without supplements at defined time points. Per well, 4 x 10^4^ thaw-and-use KDR/NFAT-RE HEK293 cells were incubated with 20 ng/mL VEGF-A and 25 μL sample for 6 h at 37 °C, 5 % CO_2_. Each hydrogel type was tested with a sample size of n = 3 for each time point. Upon binding of VEGF-A to VEGFR-2 (KDR), receptor-mediated signaling induced luminescence of the added Bio-Glow reagent which was measured with a microplate reader Infinite M200Pro (Tecan, Crailsheim, Germany). Hydrogel extracts without loaded TIMP-3 served as additional controls.

### 2.21 Cytotoxicity of hydrogel extracts

The cytotoxic potential of hydrogel extracts from GelMA, GelMA/HA_c_ and GelMA/sHA_c_ was evaluated according to ISO 10993-5 guidelines for extract-based testing. For this purpose, two 50 μL hydrogels samples were incubated in 1 mL of the respective cell culture medium at 37 °C for 72 h under sterile conditions. Following incubation, the supernatants were collected, sterile filtered (0.22 μm), and used as test solutions. This experiment was performed in triplicates per gel composition. Immortalized human microvascular endothelial cells (huMECs) were cultured in endothelial growth medium supplemented according to the supplier's instructions (INS-ME-1012, ScreenEx). NHDFs and vascular smooth muscle cells (vSMCs) were maintained in DMEM supplemented with 10 % FCS and 1% penicillin/streptomycin (P/S). 7500 cells/well were seeded in transparent 96-well plates and cultured overnight at 37 °C in a humidified incubator. On the following day, culture medium was removed and replaced with the hydrogel extracts. For control conditions, cells were cultivated in their respective culture medium without hydrogel extract. Cells were incubated for an additional 48 h. Subsequently, cell viability was assessed using the CellTiter-Blue assay (Promega) according to the manufacturer's instructions.

### 2.22 Immunological analysis

Blood samples were collected from healthy donors at the Institute of Clinical Hemostaseology and Transfusion Medicine, Saarland University. Research was approved by the local ethical committee (18/23 Alansary). Peripheral blood mononuclear cells (PBMCs) were purified using gradient centrifugation. Monocytes were isolated using magnetic separation kit (Milteyni, #130-096-537) as in Ref. [72]. Monocytes were seeded at a density of 2 x 10^6^ cells/mL and cultured for 5 days in DMEM-Glutamax (ThermoFisher Scientific 61965-026) medium containing 10 % FCS, 1 % streptomycin and penicillin without further additives for unpolarized control cells or in presence of human GM-CSF (25 ng/mL, Milteyni # 130-093-865) or M-CSF (50 ng/ml, Miltenyi # 130-096-489) for polarization into M1 or M2 macrophages, respectively. At the end of the induction phase, cells were starved by being cultured overnight in DMEM without additives. Final polarization into M1 was done by culturing the cells for further 48 h in DMEM containing 20 ng/mL interferone-γ (IFN-γ) (Miltenyi #130-096-482) and 10 pg/mL lipopolysaccharide (LPS) (InvivoGen #tlrl-3pelps) or containing 20 ng/mL interleukin-4 (IL-4) (Miltenyi, # 130-093-920) IL-13 (Miltenyi, #130-112-410) for polarization into M2. Where indicated, the final polarization medium contained extracts from GelMA, GelMA/HA_c_ and GelMA/sHA_c_. Finally, cells were harvested using TrypLE for flow cytometry analysis. Unspecific binding of antibodies was inhibited by incubating the cells with Fc receptor blocker (Human TruStain FcX, Biolegend # 422301) for 10 min at room temperature before cells were stained with corresponding antibodies for surface markers (CD11b-PerCP, CD80-AF647 and CD206-BV421 (Biolegend)) and then analyzed using flow cytometer (FACSVerse, BD).

### 2.23 Factor Xa activity assay

Factor Xa inhibition was quantified using the Biophen Heparin Anti-Xa assay (Hyphen BioMed), adapted for use in a 96-well plate. Reaction mixtures consisted of 30 μL of sample (Hep, HA_c_ and sHA_c_) combined with the kit's R1, R2, and R3 reagents, followed by the addition of 50 μL of 2 % citric acid. Measurements were carried out in 25 mM HEPES buffer (pH 7.4) containing 150 mM NaCl. Remaining Factor Xa activity was referenced to a polysaccharide-free control, which was defined as 100 %. Dose-response curves were generated using a four-parameter logistic fit, and IC_50_ values were derived when applicable.

### 2.24 Biocompatibility of the hydrogels *in-vivo*

All experiments were approved by the local authorities (Landesamt für Verbraucherschutz, Abteilung C Lebensmittel und Veterinärwesen, Saarbrücken, Germany) and were conducted in accordance with the European legislation on protection of animals (Directive 2010/63/EU) and the NIH Guidelines for the Care and Use of Laboratory Animals (http://oacu.od.nih.gov/regs/index.htm. 8th Edition; 2011). GelMA and GelMA/sHA_c_ hydrogels were prepared in silicon molds (5 mm diameter, 1.1 mm thickness) by UV light-induced photopolymerization as described above. After freeze-drying, the samples were sterilized under UV light for 15 min. The hydrogels were incubated with 50 ng TIMP-3 each in sterile physiological saline solution (0.9 % w/v NaCl in distilled water). To investigate the in-vivo function of the gels, GelMA + TIMP-3 und GelMA/sHA_c_ + TIMP3 gels were subcutaneously transplanted into the flanks of eight 14-week-old BALB/c mice (Janvier Labs, Le Genest-St-Isle, France). On day 14 after transplantation, the mice were sacrificed by cervical dislocation under anesthesia, and the gels were carefully excised and further processed for immunohistochemical analyses.

### 2.25 Immunohistochemistry

The explanted gels were embedded in OCT and 10-μm sections were cut. The sections were stained with primary antibodies against CD31 (1:100; dianova GmbH, Hamburg, Germany), CD68 (1:300; Abcam) and myeloperoxidase (MPO) (1:100; Abcam). A biotinylated goat-anti-rabbit IgG antibody (ready-to-use; Abcam) with peroxidase-labeled streptavidin (ready-to-use; Abcam) and 3-amino-9-ethylcarbazole as chromogen (Abcam) served as secondary antibodies. Sirius red staining of individual sections was performed according to standard procedures. The quantitative analysis of the sections was performed with a BX53 microscope and the imaging software cellSens Dimension (version 1.11; Olympus, Hamburg, Germany). The numbers of CD68^+^ macrophages (given in mm^2^), MPO^+^ neutrophilic granulocytes (given in mm^2^) and CD31^+^ events were assessed in 3 regions of interests (ROIs) in the border zones (granulation tissue) of each transplanted gel. The intensity of Sirius red staining was evaluated in 3 ROIs within gels as described previously [73].

### 2.26 Matrix degradation bioassay

Dentin slices were prepared from extracted human teeth. These teeth were removed for medical reasons and left behind by the patients. The patients agreed to further use of the teeth for teaching and research. The teeth were stored in collection jars completely anonymously in 0.1 % thymol. None of the teeth exhibited root caries, and all had fully formed root apices. Crowns, apical 2 mm of the roots, and root cementum were removed. The roots were segmented into 2 mm thick slices using a water-cooled preparation diamond. Specimens were stored in 1 % saline at 4 °C until enzyme activity testing. To expose collagen and activate latent MMPs, dentin slices were demineralized in 0.5 M EDTA for 10 min at room temperature with agitation. Slices were then incubated in wells of a black 96-well microplate with 100 μL of assay buffer and 20 μL of fluorescein-labeled gelatin (gelatin-FITC) solution from the EnzChek Gelatinase/Collagenase Assay Kit (Fisher Scientific, Schwerte, Germany) as described in Ref. [74] according to the manufacturer's protocol.

To determine the effects of hydrogel-released TIMP-3 on the proteolytic activity of the dentin matrix, hydrogels were loaded with 1 ng TIMP-3 per 1 μL polymer mixture in EnzChek assay buffer. After 24 and 168 h, 100 μL of the hydrogel-released TIMP-3 was added to the dentin slices prior to the addition of 20 μL gelatin-FITC. Enzymatic cleavage of the gelatin-FITC substrate was followed up by fluorescence measurements (excitation: 495 nm, emission: 515 nm). Fluorescence kinetics were measured at defined time points over the course of 120 min using a microplate reader Infinite M200Pro (Tecan, Crailsheim, Germany). Enzyme activity values (μU/mg dentin) were calculated by referencing fluorescence values to standard curves prepared with collagenase type IV from *Clostridium histolyticum* (0 - 2 μU/mL range). Enzyme activity was normalized to dentin slice weight. Sirius red staining and de-staining was used to assess the amount of degraded collagen matrix in the dentin slices 7 days after activation of dentin-bound MMPs [75]. The absorbance of Sirius red from the samples was measured at 540 nm [71]. Each hydrogel type was tested with a sample size of n = 10.

### 2.27 Ex-vivo human skin model

Body donors consented to body donation for scientific studies during their lifetime at the Anatomical Institute of Saarland University, Homburg/Saar, Germany. The ethical committee of the Medical Association of Saarland approved the study (number 162/20). Skin biopsies (Two female, median age: 89.5 ± 2.5 years, Caucasians with a skin type II-III on the Fitzpatrick scale) were collected within 24 h after death from the upper hip of the body donor from skin areas that were macroscopic without pathological findings and without signs of putrefaction [76]. The hip was sterilized using antiseptic Octeniderm (Schülke & Mayr GmbH. Norderstedt, Germany). A sterile 4.0 mm biopsy punch (SMI AG, St. Vith, Belgium, #ZBP4) was used to pre-cut a standardized circular area. The epidermis was removed using forceps and scalpel (Präzisa plus, Dahlhausen & Co. GmbH, Köln, Germany) to create a defined wound area. Afterwards, biopsies (consisting of epidermis, dermis, and a portion of subcutaneous fat) were retrieved using a sterile 10.0 mm biopsy punch (SMI AG, St. Vith, Belgium, #ZBP10), forceps and scalpel (Präzisa plus, Dahlhausen & Co. GmbH, Köln, Germany) with the 4-mm lesion in the middle of the skin-biopsy. After sampling, biopsies were immediately transferred into a 50 mL collecting tube containing cell culture medium (DMEM: Gibco, life technologies limited, United Kingdom, 10 % FSC, and 1 % streptomycin and penicillin). Under sterile conditions, biopsies were transferred to 24-well tissue culture plate (Falcon®, Corning Inc. USA, #353047) containing 250 μL cell culture medium ensuring the air-liquid interface and cultured at 37 °C and 5 % CO_2_ (Heraeus Kendro, HERAcell Inkubator). Samples were positioned so that the dermal side was in contact with medium while the epidermal surface remained exposed to air.

Before treatment, any potential liquid was removed from the wound beds using sterile cotton swabs (Boettger GmbH, Bodenmais, Germany). Wounds were then treated with 3 μL of a protease-rich inflammatory cocktail prepared in PBS containing Ca^2+^ and Mg^2+^. Each 3 μL aliquot contained 100 U CHC and 50 ng/mL recombinant human TNF-α. Hydrogel treatment was performed immediately after application of the protease cocktail. GelMA/sHA_c_ hydrogels (10 μL per wound) were applied directly into the wound bed and crosslinked in situ by UV irradiation for 30 s. Where indicated, hydrogels contained 10 μg human TIMP-3 per wound. Control conditions included (i) wounds treated with hydrogel lacking TIMP-3, and (ii) wounds without hydrogel. Culture medium was replaced daily. After 72 h of ex-vivo culture, biopsies were fixed in 4 % PFA in PBS at 4 °C for 24 h, processed for paraffin embedding according to standard histological procedures, and sectioned at 5 μm. Sections were taken from the central region of the biopsy to include the wound bed. Paraffin sections were stained with Sirius red to visualize collagen. Bright-field images were acquired using a Keyence microscope BZ-X810 (KEYENCE, Frankfurt am Main, Germany). Each section including the wound area was captured at identical exposure settings across experimental groups.

Collagen content in human skin biopsies was quantified using Sirius red staining followed by dye elution. Entire 10-mm biopsy samples from each experimental condition were processed as individual specimens (n = 8 per group). After 72 h of culture, samples were washed in PBS and fixed in 4 % PFA for 24 h. Fixed tissues were incubated in 0.1 % Sirius red in picric acid for 1 h under gentle agitation to stain fibrillar collagen. Excess dye was removed by thorough washing in 0.01 N HCl until the supernatant remained clear. For quantitative assessment, the bound dye was eluted from each biopsy using 0.1 N NaOH for 1 h. The absorbance of the resulting solution was measured at 540 nm using the microplate reader Infinite M200Pro (Tecan, Crailsheim, Germany). Absorbance values were normalized to the initial wet weight of each biopsy to account for sample-to-sample size differences.

### 2.28 Statistical analysis and software

Statistical analyses were performed using the statistical software integrated in GraphPad Prism 10 (GraphPad Software, San Diego, USA). A one- or two-way analysis of variance (ANOVA) followed by Tukey's multiple comparisons test was conducted to determine statistical differences between the groups, if not stated otherwise. Servier Medical Art by Servier, licensed under a creative common attribution 3.0 unported license was used for image generation in the graphical abstract, Fig. 6, Fig. 7. The data are represented as mean ± standard deviation.

## 3 Results

### 3.1 Sulfated HA acrylates prolong hydrogel stability

**Fig. 2:**
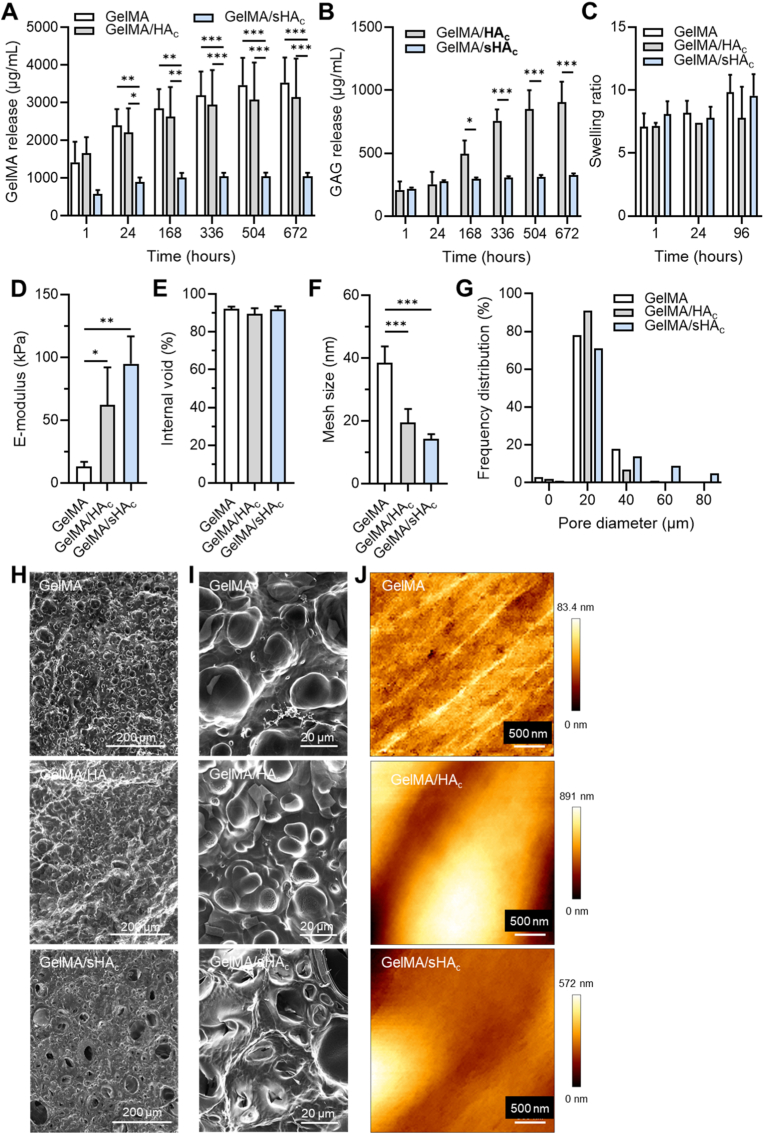
Hydrogel characteristics. The hydrogel stability was analyzed over 28 days by following the release of GelMA (A), HA_c_ and sHA_c_ (B) from the gels. The amounts of GelMA were quantified via the BCA assay, HA_c_ was determined using the hexosamine assay and sHA_c_ was quantified via the DMMB assay. The swelling ratio (C) and the compressive E-module (Young's modulus) (D) of GelMA, GelMA/HA_c_ and GelMA/sHA_c_ hydrogels were determined as further hydrogel characteristics. The E-modulus refers to the bulk compressive modulus obtained from uniaxial compression of rehydrated, freeze-dried hydrogels, calculated from the linear region (0 - 15 % strain) of the stress-strain curve. Based on the determined values from (C) and (D), the internal void and mesh size of the polymer network were calculated (E, F). The surface morphology was assessed via SEM, and the pore diameter distribution was analyzed via ImageJ (G). Images with 100× (H) and 1000× (I) magnification are shown. (J) AFM images of GelMA, GelMA/HA_c_, and GelMA/sHA_c_ hydrogels in the swollen state (measured in PBS), recorded in height min-max mode. Two-way ANOVA: ∗p < 0.05, ∗∗p < 0.01, ∗∗∗p < 0.001. One-way ANOVA for E-module: p < 0.05, ∗∗p < 0.01.

GAG-functionalized hydrogels were prepared by UV cross-linking GelMA with cross-linkable GAGs as described in Fig. 1. The hydrogel characteristics are displayed in Fig. 2. While there were no significant differences in the GelMA release after hydrogel incubation at 37 °C for 1 h, prolonged incubation showed a significantly lower GelMA release from hydrogels containing sHA_c_ compared to GelMA and GelMA/HA_c_ gels (Fig. 2 A). Biochemical analyses demonstrated the incorporation of HA_c_ and sHA_c_ into the hydrogels. Following the GAG release over the course of 28 days, analyses revealed a significantly higher HA_c_ release from the GelMA/HA_c_ gels compared to sHA_c_ from GelMA/sHA_c_ gels starting after 7 days of incubation (Fig. 2 B). No statistically significant differences between the hydrogel types over time were detected for the swelling ratio (Fig. 2C). However, the E-modulus of GAG-containing hydrogels were significantly increased compared to GelMA gels. There were no significant differences between GelMA/HA_c_ and GelMA/sHA_c_ gels (Fig. 2 D). All hydrogels exhibited a comparable porosity with an internal void fraction of approximately 90 % (Fig. 2 E). Incorporation of HA_c_ or sHA_c_ into the GelMA matrix, however, resulted in a significant reduction of the hydrogel mesh size (Fig. 2 F). Scanning electron microscopy demonstrated a comparable smooth surface for all hydrogel types with pore diameters predominantly around 20 μm (Fig. 2G–I). AFM analysis of hydrogels in the hydrated state revealed surface height variations ranging from 0 to 83.4 nm for GelMA, 0 to 891 nm for GelMA/HA_c_, and 0 to 572 nm for GelMA/sHA_c_ (Fig. 2 J).

Additional rheological measurements revealed formulation-dependent differences in the mechanical properties of in situ-crosslinked hydrogels (SI Fig. 1, SI Table 1). After UV-induced crosslinking, GelMA/HA_c_ exhibited a significantly higher storage modulus compared with both GelMA and GelMA/sHA_c_, while the latter two showed comparable G′ values (SI Fig. 1 B). Time sweep measurements confirmed a rapid gelation onset for all formulations upon UV irradiation, with GelMA/HA_c_ reaching the highest plateau storage modulus (SI Fig. 1C). Amplitude sweep analysis further indicated that GelMA/HA_c_ maintained its elastic behavior over a broader strain range, reflecting an enhanced resistance to network deformation. GelMA and GelMA/sHA_c_ transitioned earlier into non-linear viscoelastic behavior (SI Fig. 1 D). Frequency sweep data supported these observations. GelMA/HA_c_ exhibited the highest and most frequency-independent G′, whereas GelMA and GelMA/sHA_c_ displayed comparable G′ (SI Fig. 1 E). Together, these data demonstrate that HA_c_ incorporation reinforces the GelMA network, while sHA_c_ incorporation maintains shear stiffness comparable to GelMA.

In addition, we assessed the degradability of the hydrogels in-vitro. All hydrogel formulations were degradable using a collagenase/hyaluronidase mixture (SI Fig. 2). After 1 h, GelMA/HA_c_ hydrogels showed a delay in GelMA release compared with GelMA hydrogels, whereas GelMA and GelMA/sHA_c_ displayed similar early degradation. Both HA_c_ and sHA_c_ were rapidly liberated, with roughly 60 - 70 % detected after 1 h and near-complete degradation after 24 h (SI Fig. 2 A). Agarose gel electrophoresis of the released GAGs showed a broad blue band for GelMA/HA_c_ after 24 h, comparable in height to the HA_c_ standard and spanning higher and lower molecular weight regions, consistent with partially crosslinked HA_c_ and smaller degradation products. GelMA/sHA_c_ hydrogels produced a very broad, intense purple band ranging from the low–molecular weight HA standard to the sHA_c_ standard, indicating partially retained sHA_c_ crosslinks and a wide molecular weight distribution (SI Fig. 2 B).

### 3.2 GAG functionalization tunes the TIMP-3 release from the hydrogels

**Fig. 3:**
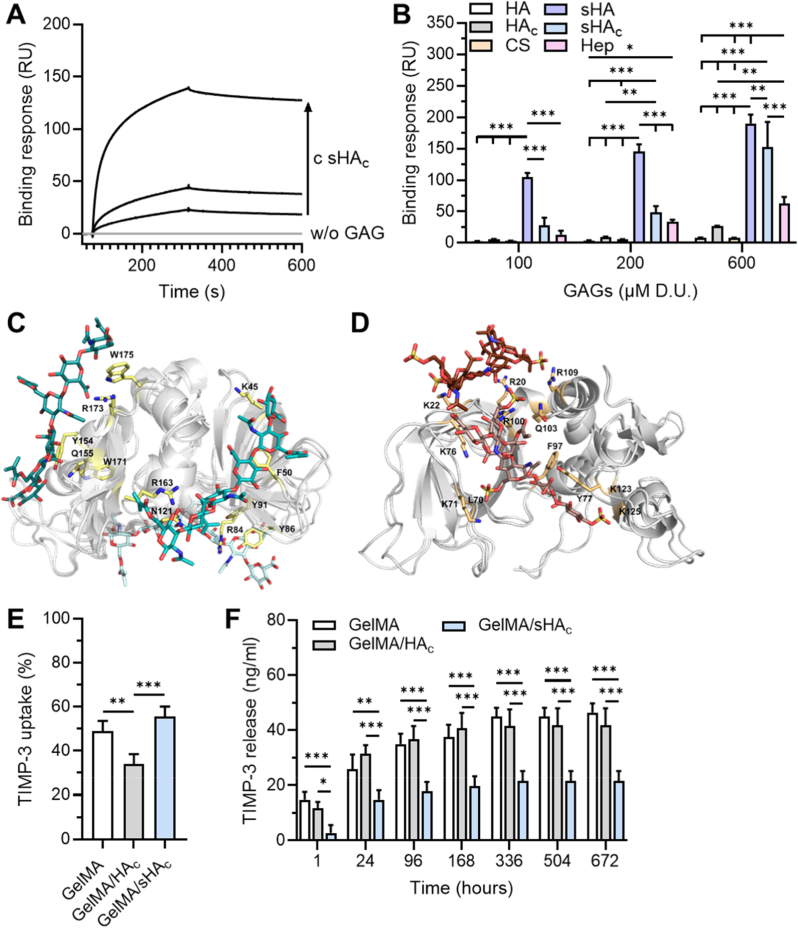
**TIMP-3 binding and release characteristics of the hydrogels.** Surface plasmon resonance analysis of GAG binding to immobilized TIMP-3. (A) Representative sensorgrams of sHA_c_ binding to TIMP-3 are shown. (B) Binding responses of native GAGs (HA, CS, Hep) in comparison to chemically modified HA derivatives. (C, D) Predicted binding of methacrylated and acrylated GAG derivatives (atom-colored sticks in cyan and brown gradient, respectively) to TIMP-3 (grey cartoon). MD-refined structures from the 20 ns of simulations of the (C) HA_c_/TIMP-3 and (D) sHA_c_ (*i.e.* HA6_3AC1)/TIMP-3 complexes are shown. In panel D, TIMP-3 has been rotated (i.e. 20° in x and 170° in y) in order to better show the GAG binding. TIMP-3 residues interacting with HA_c_ and sHA1_c_ are labeled and represented in yellow and orange sticks, respectively. (E, F) Hydrogels were loaded with TIMP-3 in SBF. The amount of non-bound and released TIMP-3 from the hydrogels in SBF was quantified by ELISA. One-way ANOVA for TIMP-3 uptake; two-way ANOVA for TIMP-3 release: ∗p < 0.05, ∗∗p < 0.01, ∗∗∗p < 0.001.

The influence of HA functionalization on its interaction with TIMP-3 was evaluated by surface plasmon resonance (SPR) (Fig. 3A and B). Non-sulfated HA (i.e. HA, HA_c_) and native low-sulfated CS exhibited no or only marginal binding responses. Introduction of sulfate groups to HA significantly increased the binding strength to TIMP-3. The highest binding levels were detected for sHA lacking additional crosslinkable functionalities. However, sHA_c_ still showed a concentration-dependent binding to TIMP-3, with signal intensities comparable to those obtained with native Hep.

Recognition of hexasaccharide HA_c_ (*i.e.* HA_6 MC) and sHA_c_ (*i.e.* HA6_2AC1 and HA6_3AC1) by TIMP-3 was investigated by means of blind docking. MD simulations were used to refine the obtained GAG binding poses (Fig. 3C and D) and calculate free energies of binding and per-residue energic contributions. The obtained energies indicated more favorable binding of sHA_c_ derivatives to TIMP-3 than HA_c_ (Table 2).

**Table 2:** MM-GBSA free energies of binding^a^ of methacrylated and acrylated GAG derivatives to TIMP-3, and list of interacting residues.

GAG variant	Free energy of binding ΔG (kcal/mol)	Interacting residues
HA_6 MC dp6 (cluster1)	−25.0 ± 4.1	L106, Y154, Q155, W171, R173, W175
HA_6 MC dp6 (cluster2)	−22.1 ± 2.6	K45, F50, R84, N121, R163
HA_6 MC dp6 (cluster3)	−32.4 ± 1.0	R84, Y86, Y91

HA6_2AC1 dp6 (cluster1)	−32.2 ± 3.5	C3, N14, L67, R84, Y86, C95, N96, K125, S126, Y128
HA6_2AC1 dp6 (cluster2)	−32.9 ± 4.8	R48, G49, F50, M51, K52, R84, K89, K123, N138, R163
HA6_2AC1 dp6 (cluster3)	−26.7 ± 11.0	K45, R48, G49, F50, T51, K52, R84 K123

HA6_3AC1 dp6 (cluster1)	−49.5 ± 13.4	P5, L70, K71, K76, Y77, F97, V98, R100, Q103, Y151
HA6_3AC1 dp6 (cluster2)	−34.6 ± 9.5	R20, K22, Y77, R109, K123, K125

a Values represent the mean ± SD from three independent 40 ns MD simulations.

The TIMP-3 binding capacity of the hydrogels was assessed by incubating different hydrogel formulations with defined concentrations of TIMP-3. GelMA and GelMA/sHA_c_ hydrogel groups demonstrated comparable binding efficiency, retaining approximately 50 % of the soluble TIMP-3 regardless of the presence of functionalized sHA_c_ (Fig. 3 E). However, hydrogels containing HA_c_ bound significantly less TIMP-3 than the other two groups. The release kinetics of hydrogel-bound TIMP-3 were monitored over a 28-day period, demonstrating a continued release of TIMP-3 with significant differences in the amount of released TIMP-3 between the hydrogel types for all time points, except for the 1 h time point (Fig. 3 F). The amount of hydrogel-released TIMP-3 decreased in the following order: GelMA ≈ GelMA/HA_c_ > GelMA/sHA_c_. Hydrogels containing sHA_c_ released significantly less TIMP-3 compared to GelMA and GelMA/HA_c_ hydrogels. Immunofluorescence imaging further demonstrated that TIMP-3 predominantly localized within the peripheral regions of all hydrogel formulations (SI Fig. 3).

### 3.3 sHA-functionalization of hydrogels enhances the sustained TIMP-3 bioactivity

**Fig. 4:**
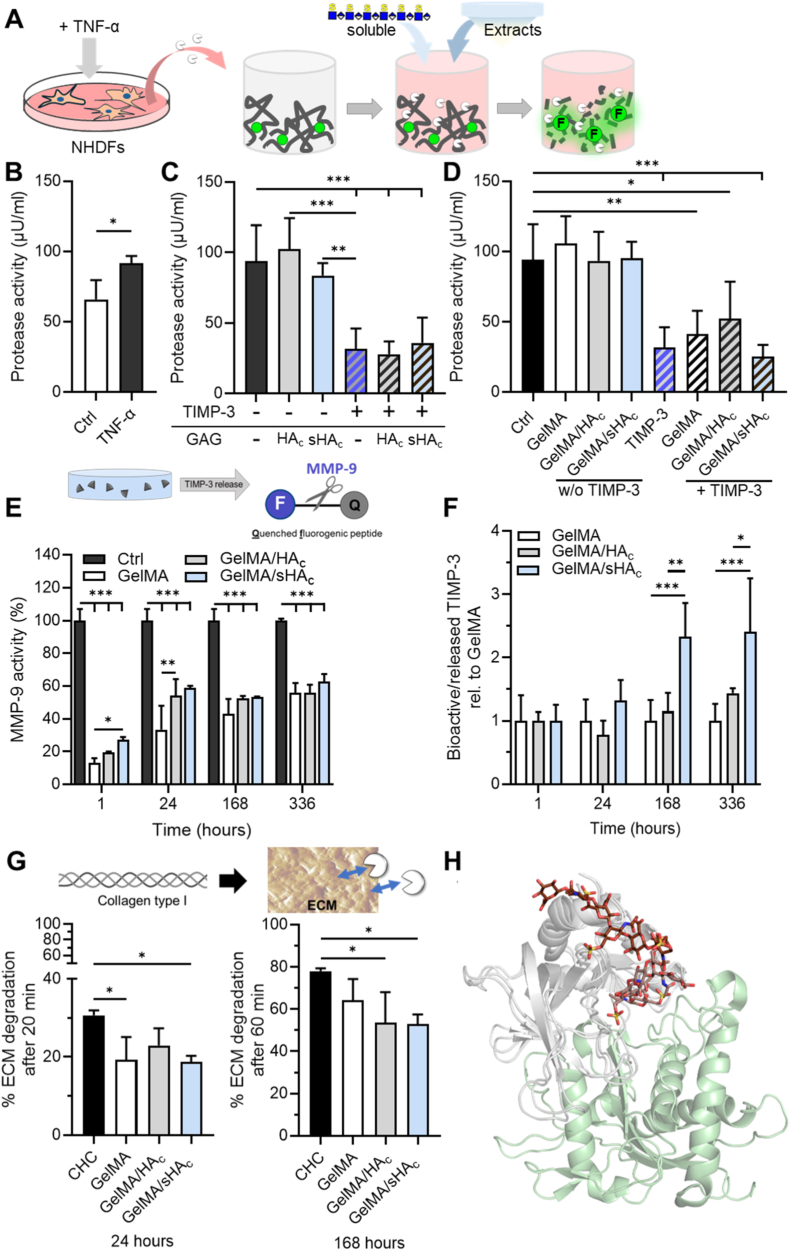
**TIMP-3 maintains protease inhibitory activity in the presence of sHA_c_ and hydrogels release bioactive TIMP-3.** (A-D) Influence of soluble GAGs and hydrogel extracts on TIMP-3-mediated inhibition of protease activity in TNF-α-stimulated NHDFs. (A) Schematic of the experimental design. Inflammation was modeled by stimulating NHDFs with TNF-α, inducing increased protease secretion. Gelatinase/collagenase activity in supernatants was quantified using the EnzChek assay with a fluorogenic gelatin substrate in the presence or absence of soluble TIMP-3, soluble GAGs or hydrogel extracts. (B) Protease activity in the supernatants after TNF-α treatment relative to unstimulated controls. (C) Protease activity of TNF-α-stimulated supernatants incubated with soluble GAGs (HA_c_, sHA_c_) with or without TIMP-3. (D) Protease activity of TNF-α-stimulated supernatants incubated with hydrogel extracts (prepared by 72 h hydrogel incubation in medium) in the absence or presence of TIMP-3. One-way ANOVA: ∗p < 0.05, ∗∗p < 0.01, ∗∗∗p < 0.001. Only significant differences relative to the Ctrl without TIMP-3 or relative to TIMP-3 alone are shown in C/D. (E) The inhibitory potential of TIMP-3 released from the hydrogels was measured using a MMP-9 activity assay. (F) The ratio of bioactive TIMP-3 to the total amount of released TIMP-3 was calculated and expressed as a fold change relative to GelMA hydrogels without GAGs. (G) Collagen-based ECMs were incubated with collagenase (CHC) for 20 or 60 min with TIMP-3 released from the hydrogels after 24 or 168 h. The remaining collagen was detected after Sirius red staining and elution. Two-way ANOVA for A, B: ∗p < 0.05, ∗∗p < 0.01, ∗∗∗p < 0.001. One-way ANOVA for C: ∗p < 0.05. (H) Molecular rationale for the regulatory role of sHA_c_ on TIMP-3-mediated protease inhibition. The MD-refined complex of TIMP-3 (in grey) with HA6_3AC1 (atom-colored brown sticks, color gradient as in Fig. 3 D) is shown superimposed with the TIMP-3/ADAM complex (PDB ID 3CKI). ADAM is shown in green, and the corresponding TIMP-3 structure has been omitted for clarity.

The potential influence of soluble GAGs and hydrogel-derived extracts on the TIMP-3-mediated inhibition of protease activity in TNF-α–stimulated NHDFs was evaluated (Fig. 4A–D). Soluble HA_c_ and sHA_c_ alone did not alter protease activity, whereas TIMP-3 induced a significant reduction (Fig. 4C). Of note, TIMP-3 inhibited the protease activity to a similar extent in the presence or absence of HA_c_ or sHA_c_, demonstrating that its inhibitory capacity is preserved in the presence of soluble GAGs. Hydrogel extracts obtained after 72 h of incubation were also assessed for potential interference with TIMP-3 function (Fig. 4 D). Consistent with the soluble GAG data, the extracts alone had no effect on protease activity, and their combination with TIMP-3 resulted in a level of protease inhibition comparable to TIMP-3 alone.

The bioactivity of hydrogel-released TIMP-3 was assessed by measuring the inhibitory potential of the protein against MMP-9. Hydrogel-released TIMP-3 up to 14 days after initial protein loading significantly decreased the MMP-9 proteolytic activity (Fig. 4 E). TIMP-3 released from GelMA after 1 h reduced the MMP-9 activity significantly stronger than TIMP-3 released from GelMA/sHA_c_. However, at later time points, there were no significant differences between the different hydrogel types except for GelMA and GelMA/HA_c_ 24 h after loading with TIMP-3. A comparison of the ratios of bioactive TIMP-3, based on its MMP-9 inhibitory activity, to the total amount of TIMP-3 released from each hydrogel type demonstrated that TIMP-3 bioactivity was comparable across all hydrogel groups after 1 h and 24 h of release (Fig. 4 F). However, at later time points, TIMP-3 released from GelMA/sHA_c_ had a significantly higher bioactivity compared to TIMP-3 from hydrogels without sulfated GAGs.

The effects of released TIMP-3 on matrix degradation were analyzed in-vitro using a collagen-based ECM model (Fig. 4 G). TIMP-3 released after 24 h and 7 days from GelMA/sHA_c_ gels significantly decreased matrix degradation compared to collagenase-treated controls. Moreover, TIMP-3 released from GelMA after 24 h and GelMA/HA_c_ after 7 days significantly reduced matrix degradation.

We also used molecular modeling to investigate whether sHA_c_ (i.e. HA6_3AC1) could affect TIMP-3 recognition by MMP. The structural superimposition of the MD-refined HA6_3AC1/TIMP-3 complex with the structure of ADAM/TIMP-3 (PDB ID 3CKI) (Fig. 4H) suggested no competition between HA6_3AC1 and ADAM for the same TIMP-3 recognition site. Therefore, sHA_c_ should not affect the inhibitory role of TIMP-3 on metalloproteinases.

### 3.4 sHA_c_ modulates TIMP-3 anti-angiogenic activity by restoring VEGF signaling and microvessel sprouting

**Fig. 5:**
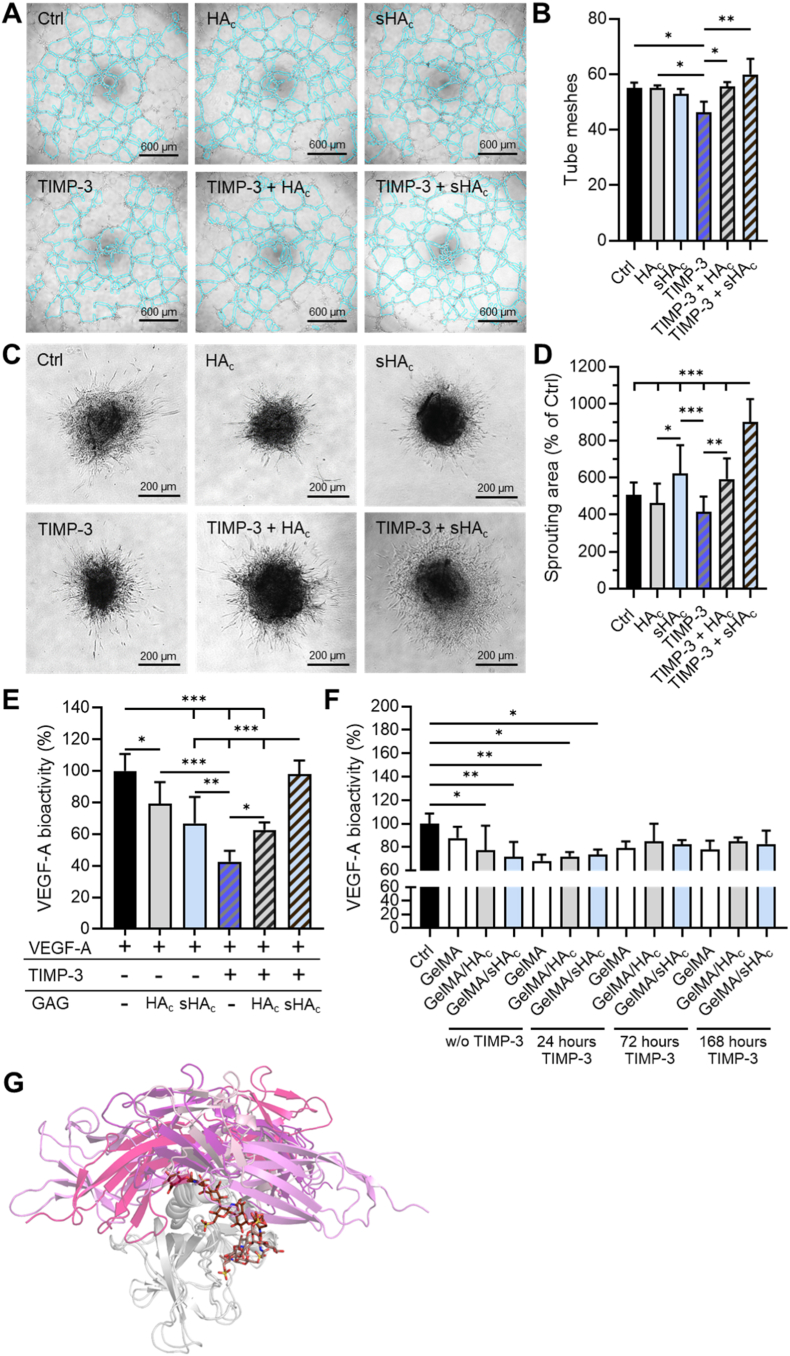
**sHA_c_ attenuates the anti-angiogenic potential of TIMP-3.** (A, B) Tube formation assay with HUVECs in the presence or absence of soluble HA_c_, sHA_c_, and/or TIMP-3. Treatments were applied at seeding. After 6 h of incubation, (A) representative microscopic images were acquired, and (B) tube meshes were quantified by ImageJ. (C, D) MVF spheroid sprouting assay. Spheroids were treated once after embedding with or without soluble HA_c_, sHA_c_, and/or TIMP-3. (C) Representative microscopic images are shown. (D) Sprouting area was quantified relative to vehicle control. (E) VEGF-A bioactivity in the presence or absence of soluble HA_c_, sHA_c_, and/or TIMP-3 assessed using a VEGFR-2 reporter cell line. (F) Bioactivity of TIMP-3 released from the hydrogels evaluated in the VEGFR-2 reporter assay compared to hydrogels extracts lacking TIMP-3. One-way ANOVA: ∗p < 0.05, ∗∗p < 0.01, ∗∗∗p < 0.001. (G) Molecular rationale for the regulatory role of sHA_c_ on the anti-angiogenic effects of TIMP-3. The MD-refined complex of TIMP-3 (in grey) with HA6_3AC1 (atom-colored brown sticks, color gradient as shown in Fig. 3 D) superimposed with four of the top ten TIMP-3/VEGFR-2 docking poses. The four VEGFR-2 molecules are shown in different pink gradients representing each docking pose, and the four corresponding TIMP-3 structures have been omitted for clarity.

Since TIMP-3 is a known VEGF-A competitor, its anti-angiogenic activity in the presence of soluble GAGs and hydrogel extracts was further analyzed. HUVEC tube formation was significantly reduced by TIMP-3, while HA_c_ and sHA_c_ alone did not alter network formation (Fig. 5A and B). The combination of TIMP-3 with HA_c_, and especially with sHA_c_, restored the number of tube meshes to values comparable to the control. Analysis of angiogenesis-related proteins in HUVECs revealed that TIMP-3 reduced the pro-angiogenic mediator angiogenin, while increasing anti-angiogenic or matrix-associated proteins including thrombospondin-1 and endostatin (SI Fig. 4). sHA_c_ treatment led to lower detection of angiogenic factors such as angiopoietin-2, FGF-4, VEGF, vasohibin. Co-treatment with sHA_c_ and TIMP-3 restored factors including angiogenin and thrombospondin-1 to near-control levels.

**Fig. 6:**
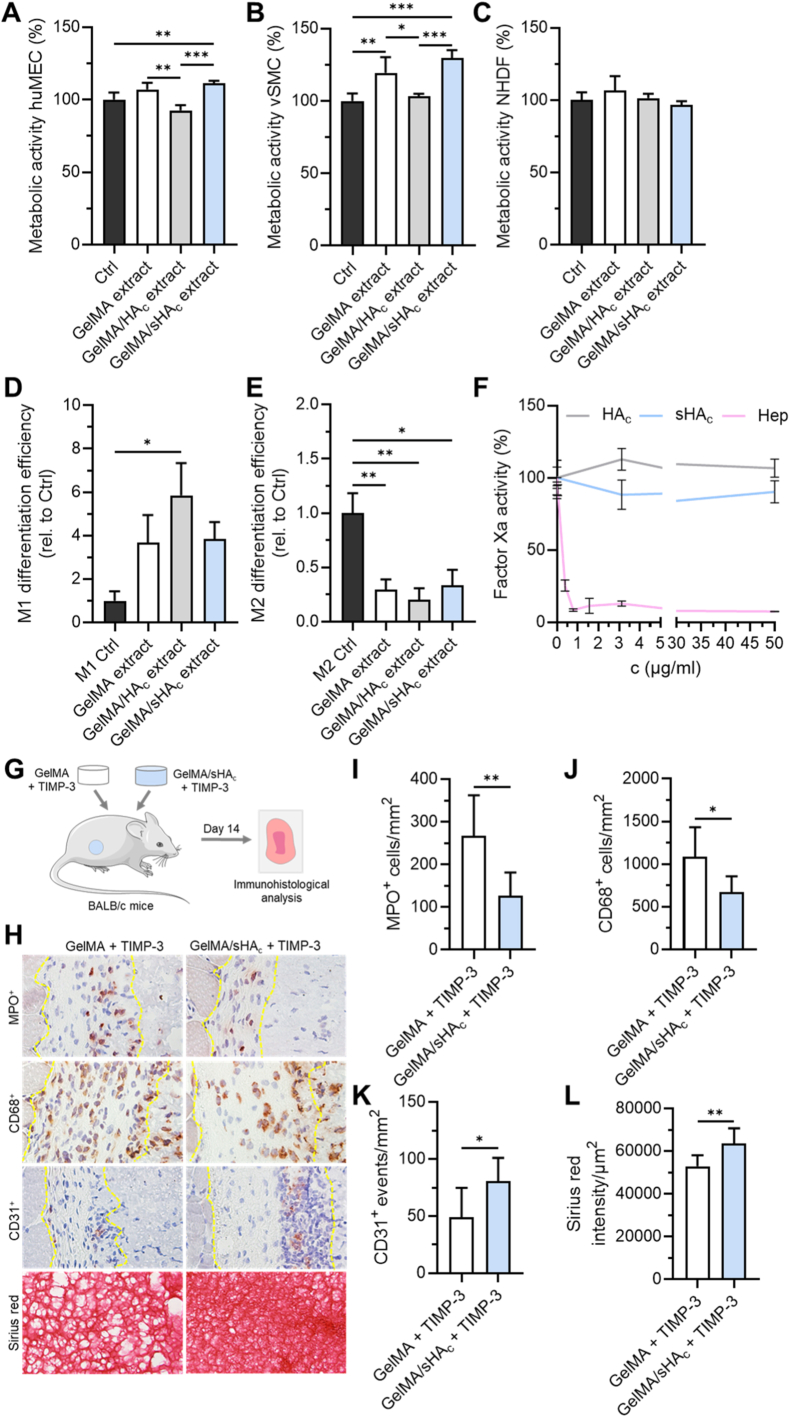
Biocompatibility of hydrogels. (A-C) Hydrogels were incubated in the respective cell culture media for 72 h, and the obtained extracts were used to assess their effects on the metabolic activity of huMECs (A), vSMCs (B), and NHDFs (C) after 48 h of culture. (D, E) Hydrogel extracts were added to primary human monocytes obtained from five independent donors. The differentiation efficiency of these immune cells into M1 (D) or M2 (E) macrophages was analyzed by flow cytometry using specific markers. (F) Anti-factor Xa activity of HA_c_ and sHA_c_ was determined in comparison with Hep using a chromogenic assay. (A-F) One-way ANOVA: ∗p < 0.05, ∗∗p < 0.01, ∗∗∗p < 0.001. (G) In-vivo assessment of GelMA and GelMA/sHA_c_ hydrogels loaded with TIMP-3. Experimental overview: TIMP-3-loaded GelMA and GelMA/sHA_c_ hydrogels were implanted subcutaneously into BALB/c mice for 14 days. (H) Representative histological images of explanted gels stained for MPO (neutrophils), CD68 (macrophages), CD31 (microvessels), and Sirius red (collagen deposition). The granulation tissue between the muscle tissue and the implant is highlighted by dotted yellow lines. (I-L) Quantification of MPO^+^ and CD68^+^ cells, CD31^+^ events, and Sirius red intensity (three ROIs per sample). Statistical analysis was performed using an unpaired *t*-test with Welch's correction: ∗p < 0.05, ∗∗p < 0.01.

**Fig. 7:**
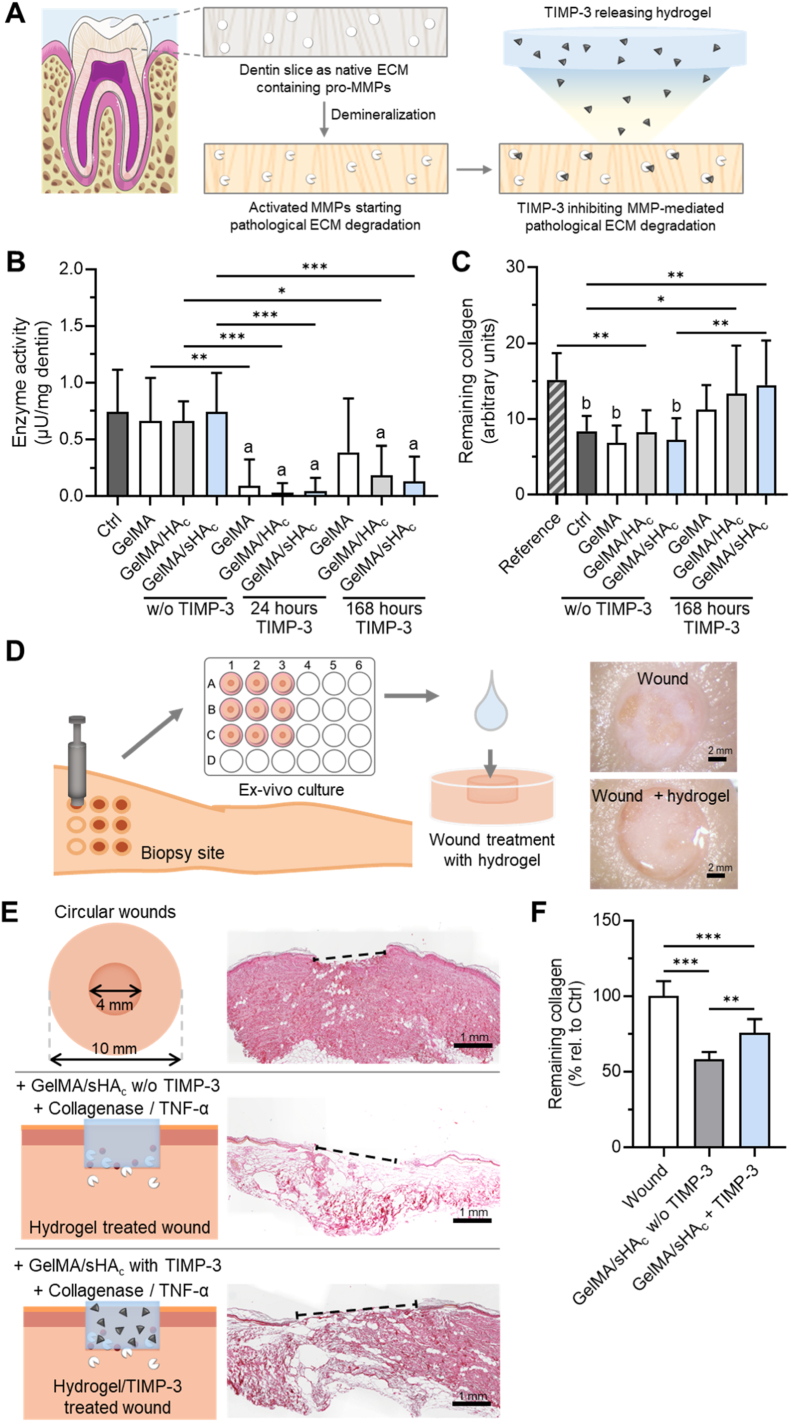
**TIMP-3 from GelMA/sHAc hydrogels reduces matrix degradation ex-vivo.** (A) Schematic overview of the ex-vivo matrix degradation assay. Human dentin slices were demineralized to expose the collagen matrix and activate endogenous proteases and subsequently incubated with TIMP-3-containing or TIMP-3-free hydrogels to assess their effects on native matrix degradation. (B) The potential of TIMP-3 released from hydrogels after 24 and 168 h to influence native matrix turnover was analyzed in an ex-vivo dentin slice model using the EnzChek assay, in comparison to hydrogels without TIMP-3. Demineralized dentin slices without additional treatment served as controls (Ctrl). (C) Collagen degradation by endogenous matrix-located proteases was assessed in the presence or absence of hydrogel released TIMP-3 or hydrogels without TIMP-3. Collagen preservation was measured by Sirius red staining followed by dye elution. Inactivated dentin slices from autoclaved teeth served as reference. One-way ANOVA: ∗p < 0.05, ∗∗p < 0.01, ∗∗∗p < 0.001., For (A) a = ∗∗∗p < 0.001 vs. Ctrl, for (B) b = ∗∗∗p < 0.001 vs. Reference. Significant differences were determined versus Ctrl/Reference and between hydrogels with or without TIMP-3 of the same composition to distinguish material-from TIMP-3-specific effects. (D, E) TIMP-3-loaded GelMA/sHA_c_ hydrogels reduce ECM degradation in a human ex-vivo skin model. (D) Schematic of the ex-vivo human skin model. 10 mm skin biopsies with a 4 mm wound were collected within 24 h post-mortem and cultured ex-vivo. Pathological ECM degradation was induced by collagenase and TNF-α treatment. GelMA/sHA_c_ hydrogels with or without TIMP-3 were applied to the wound surface and cultured for 72 h. (E) Overview of sample groups and representative Sirius red-stained histological sections showing ECM organization: intact wound (no pathological ECM degradation), wound with induced ECM degradation treated with GelMA/sHA_c_, or GelMA/sHA_c_ + TIMP-3. The wound region is indicated by a dotted line. (F) Remaining collagen content quantified by Sirius red staining of human skin samples, followed by dye elution and absorbance measurement. Absorbance values were corrected for initial sample weight differences. One-way ANOVA: ∗∗p < 0.01, ∗∗∗p < 0.001.

In addition, we analyzed the effects of the treatments on MVF spheroid sprouting as 3D microtissues (Fig. 5C and D). MVF spheroids, composed of intact microvascular fragments, provide a physiologically relevant ex-vivo angiogenesis model that recapitulates essential steps of microvessel sprouting. Spheroids treated with sHA_c_ exhibited a greater sprouting area compared to those treated with HA_c_. Notably, the combination of TIMP-3 and sHA_c_ resulted in the largest sprouting area after 3 days, significantly exceeding that of TIMP-3 alone, TIMP-3 with HA_c_, and the inhibitor-free control. To assess early VEGF signaling events, we examined the short-term effects of TIMP-3 and GAGs on VEGF-A-induced VEGFR-2 activation using a VEGF bioassay (Fig. 5 E). HA_c_, sHA_c_, and TIMP-3 each significantly reduced VEGF-A bioactivity. However, the combination of TIMP-3 with sHA_c_ restored VEGF-A bioactivity to control levels. We further evaluated the influence of hydrogel extracts on VEGF-A bioactivity using the same VEGFR-2 reporter cells (Fig. 5 F). Extracts from GelMA/HA_c_ and GelMA/sHA_c_ hydrogels lacking TIMP-3 significantly suppressed VEGF-A-induced VEGFR-2 activation. TIMP-3 released within 24 h from these hydrogels reduced VEGF-A-induced receptor activation by 21 – 31 %. At later time points, however, the effect diminished. TIMP-3 released after 72 h or 168 h no longer significantly altered VEGF-A bioactivity, irrespective of hydrogel composition.

In order to gain further insights on how sHA_c_ might modulate the anti-angiogenic effects of TIMP-3, we conducted additional docking-based investigations using a previously reported 3D model of VEGFR-2 [59]. The obtained results suggested that sHA_c_ might prevent TIMP-3 binding to VEGFR-2, as sHA_c_ and VEGFR-2 appear to compete for the same recognition site (Fig. 5 G). This might explain the diminished anti-angiogenic activity of TIMP-3 in the presence of sHA_c_.

### 3.5 Biocompatibility profiles of hydrogels

To assess the biological impact of soluble components released from the hydrogels, we evaluated how the extracts affected the metabolic activity of cell types relevant to wound healing, including endothelial cells, vascular smooth muscle cells, and fibroblasts (Fig. 6A–C). Extracts from GelMA/sHA_c_ hydrogels significantly increased the metabolic activity of huMECs and vSMCs, while GelMA extracts without GAGs also enhanced vSMC activity. In contrast, none of the hydrogel extracts altered the NHDF metabolic activity (Fig. 6C).

The immunomodulatory effects of the hydrogels were evaluated by quantifying monocyte differentiation toward pro-inflammatory M1 and anti-inflammatory M2 macrophages (Fig. 6D and E). Extracts from GelMA/HA_c_ hydrogels significantly increased M1 polarization, whereas extracts from GelMA and GelMA/sHA_c_ hydrogels did not affect M1 differentiation efficiency. All extracts reduced M2 polarization relative to the control. However, this reduction was less pronounced for extracts from GelMA/sHA_c_ (Fig. 6 E).

Because chemical modification of HA may confer anticoagulant properties, the anti-factor Xa activity of HA_c_ and sHA_c_ was compared with Hep (Fig. 6 F). Hep showed dose-dependent inhibition of factor Xa with an IC_50_ of 0.26 μg/mL, whereas neither HA_c_ nor sHA_c_ displayed detectable inhibitory activity.

Since GelMA/HA_c_ hydrogels were found to stimulate a pro-inflammatory M1 macrophage polarization, this hydrogel formulation was excluded from subsequent in-vivo biocompatibility assessments. Because the early inflammatory and regenerative events within peri-implant granulation tissue are key determinants of hydrogel biocompatibility, we next examined the host response to TIMP-3-loaded GelMA and GelMA/sHA_c_ hydrogels in-vivo (Fig. 6 G). In accordance with the principle of the 3Rs, this analysis was restricted to these groups as most informative material configurations. Fourteen days after subcutaneous implantation, immunohistochemistry revealed a markedly attenuated inflammatory reaction in the GelMA/sHA_c_ group. Quantitative evaluation demonstrated significantly fewer MPO ^+^ neutrophils and CD68^+^ macrophages per mm^2^ in the granulation tissue of GelMA/sHA_c_ + TIMP-3 compared with GelMA + TIMP-3 hydrogels (Fig. 6I and J). In parallel, indicators of neovascularization and matrix remodeling were elevated in the GelMA/sHA_c_ group. The number of CD31^+^ microvessels was significantly higher, and Sirius red staining intensity was increased in the granulation tissue surrounding GelMA/sHA_c_ + TIMP-3 implants compared to GelMA + TIMP-3 (Fig. 6K and L). Together, these results demonstrate that sHA_c_ modification of TIMP-3-loaded GelMA hydrogels is associated with altered inflammatory cell presence and changes in vascular- and collagen-related histological parameters within the peri-implant granulation tissue.

### 3.6 Hydrogel-delivered TIMP-3 decreases proteolytic matrix degradation

The capacity of hydrogel-delivered TIMP-3 to mitigate proteolytic degradation of the native ECM was evaluated using an ex-vivo dentin slice model (Fig. 7). Dentin, located beneath the enamel and cementum, is characterized by a collagen-rich matrix with inherent proteolytic activity. Demineralization of dentin activates endogenous proteases, such as MMPs, initiating pathological matrix degradation, which can be monitored by adding fluorophore-labeled gelatin to the dentin slices [74] (Fig. 7 A). TIMP-3 delivered from the GelMA, GelMA/HA_c_ and GelMA/sHA_c_ hydrogels for 24 h significantly reduced the proteolytic activity of the native matrix, whereas extracts from hydrogels lacking TIMP-3 had no detectable effect (Fig. 7 A). After 7 days, no significant inhibitory effects were observed for TIMP-3 released from pure GelMA samples compared to the control. However, the enzymatic activity within the matrix incubated with TIMP-3 delivered from GelMA/HA_c_ and GelMA/sHA_c_ hydrogels after 168 h was still significantly lower compared to control samples without MMP inhibitor.

Collagen preservation within the matrix after dentin-mineralization in the absence or presence of hydrogel-released TIMP-3 was assessed via collagen-specific staining with the dye Sirius red (Fig. 7 B). Inactivated dentin slices served as a reference lacking endogenous protease activity. Activated proteolysis resulted in a marked reduction of collagen in all samples without TIMP-3, irrespective of hydrogel presence. No significant differences of GelMA released TIMP-3 on the collagen levels were observed compared to the control with pathological ECM degradation or activated dentin matrix treated with GelMA without TIMP-3. TIMP-3 released from GelMA did not improve collagen retention compared with either the degraded control or GelMA alone. Similarly, TIMP-3 released from GelMA/HA_c_ did not significantly alter collagen levels relative to its respective hydrogel control. In contrast, dentin matrices treated with TIMP-3 released from GelMA/sHA_c_ hydrogels retained a significantly higher collagen content than untreated controls and GelMA/sHA_c_ samples lacking TIMP-3 (Fig. 7 B).

In addition, the potential of TIMP-3-loaded GelMA/sHA_c_ hydrogels to reduce pathophysiological ECM degradation was evaluated using a human ex-vivo skin model (Fig. 7D–SI Fig. 5). Representative histological skin sections were stained with Sirius red to visualize collagen and ECM organization after 72 h of culture (Fig. 7 E). Wounded skin without induced pathophysiological degradation displayed an intense red staining of the dermis, indicating a dense collagen structure, along with lighter regions corresponding to adipose tissue. In contrast, wounds with induced ECM degradation treated with GelMA/sHA_c_ hydrogels showed a loosened tissue architecture and qualitatively reduced red staining intensity, reflecting collagen loss. Notably, wounds treated with TIMP-3-loaded GelMA/sHA_c_ hydrogels exhibited a more compact ECM structure and stronger red staining compared to the GelMA/sHA_c_-treated samples without TIMP-3, indicating reduced ECM degradation. Induced pathological ECM degradation resulted in a significant reduction of collagen content in GelMA/sHA_c_-treated wounds lacking TIMP-3 compared to intact wounded skin, whereas incorporation of TIMP-3 into the hydrogel markedly preserved collagen levels relative to the TIMP-3-free hydrogel treatment (Fig. 7 F).

## 4 Discussion

MMPs play a critical role in various pathological conditions characterized by excessive matrix degradation. In this study, we designed an injectable bio-inspired hydrogel system composed of GelMA, sHA_c_ and TIMP-3 to modulate dysregulated ECM turnover and promote a shift toward physiological matrix remodeling. GelMA was chosen as substrate due to its close resemblance of collagen as main structural protein within the native ECM with its inherent MMP-cleavable regions. In addition, we synthesized sHA_c_ to covalently incorporate sHA into the GelMA hydrogel. Due to its affinity for TIMP-3, the negatively charged polysaccharide sHA_c_ was used to mimic the native TIMP-3/ECM interplay and to reduce passive diffusion of TIMP-3 from the gels.

Crosslinking sHA_c_ into the GelMA matrix enhanced the hydrogel stability over time. In line with this, sHA was found to increase the stability of collagen-based scaffolds [77]. The presence of both HA_c_ and sHA_c_ within the hydrogels significantly increased the E-modulus of GelMA-based gels. Likewise, incorporating HA_c_ into GelMA formulations was found to increase the E-modulus by 2- to 6-fold, depending on the amount of HA added [78]. In contrast, Heinemann et al. [79] reported no significant differences when comparing GelMA and GelMA/HA_c_ hydrogels. This discrepancy is likely attributable to the considerably lower HA_c_ concentrations used in their formulations, which were more than threefold lower than those employed in the present study. Moreover, the molecular weight of HA_c_ represents an additional parameter that can influence stiffness. However, the authors did not report the molecular weight of the HA_c_ used. Furthermore, this study utilized 5 % GelMA, in contrast to the 10 % GelMA employed here, highlighting the substantial influence of the GelMA:HA_c_ ratio in modulating the hydrogel's E-modulus.

Comparison of rheological and compressive data revealed that the higher compressive moduli of GelMA and GelMA/HA_c_ after rehydration can be largely explained by increased polymer volume fractions due to swelling. In contrast, the disproportionately high compressive modulus of GelMA/sHA_c_, despite a shear storage modulus similar to pure GelMA, cannot be accounted for by swelling alone. This divergence aligns with literature showing that ionic strength, counter-ions and electrostatic interactions can alter hydrogel behavior under compression far more than under shear [80]. Here, the likely mechanism is that sulfate groups in sHA_c_ form ionic bridges with cations in the culture medium and engage in electrostatic/hydrogen bonds with the gelatin network, thereby introducing additional physical crosslinks that reinforce the network in bulk compression but are less detectable by small-strain shear rheology. Within the viscoelastic regime probed by oscillatory shear rheology, however, we did not observe a systematic relationship between the viscoelastic response under increasing strain and frequency and TIMP-3 binding, release or inhibitory activity.

SEM of freeze-dried gels revealed only minor morphological differences, whereas AFM analysis of swollen hydrogels showed that incorporation of HA_c_ or sHA_c_ increases surface roughness, indicating a more heterogeneous microstructure. Previously, a comparable surface roughness for pure 10 % GelMA hydrogels was reported [81]. Furthermore, the surface water absorption degree of GelMA-based composite hydrogels was linked to surface roughness changes [82]. Notably, the calculated mesh size of the hydrogels ranged from about 15 to 39 nm, which is in line with previous reports for 10 % GelMA hydrogels [83]. Given the predicted hydrodynamic diameter of TIMP-3 of about 5 nm [84], the protein would be expected to diffuse through the network without significant steric hindrance. Accordingly, steric contributions to TIMP-3 retention are expected to be minor, and differences observed in protein loading and release are more likely driven by affinity-based interactions introduced by HA_c_ or sHA_c_ rather than by a direct size-matching relationship between the protein and the mesh. A similar separation of structural and mechanical contributions has been highlighted by Wen et al., who demonstrated that pore sizes between 20 and 100 nm do not sterically limit biomolecule transport, and by Yin et al., who showed that integrating shear rheology with bulk mechanical testing is essential to decouple network architecture from functional protein interactions [85,86].

TIMP-3 as natural protease inhibitor was chosen for local delivery because of its distinctive ability to bind to the ECM. Previous studies found a sulfation-dependent interaction of TIMP-3 with native GAGs and GAG derivatives [19,37,57]. SPR measurements further confirmed concentration-dependent binding of native Hep, sHA, and sHA_c_ to TIMP-3, while non-sulfated HA and CS showed only marginal binding responses. Acrylation of the sHA backbone decreased the binding capacity of sHA_c_ relative to sHA. Similar effects, attributed to steric alterations introduced by acrylate groups in the GAG backbone, have been reported for other GAG-binding proteins [33]. However, despite its lower sulfation, sHA_c_ bound TIMP-3 at levels comparable to native Hep. As expected for diffusion-mediated loading into pre-formed hydrogels, confocal z-scanning revealed a pronounced peripheral enrichment of TIMP-3 with detectable penetration into the gel interior, reflecting the interplay between its inward diffusion through a permissive mesh and its affinity-driven retention within the outer network regions. In line with the SPR binding characteristics, the degree of GAG sulfation significantly influenced the TIMP-3 release from the developed GAG-functionalized hydrogels, with higher HA sulfation slowing release rates, thereby enabling sustained TIMP-3 delivery for up to 28 days. Consistent with this observation, our molecular models indicated that TIMP-3 interactions were stronger with sHA_c_ than with HA_c_, particularly when sHA_c_ was acrylated at position C3’. The identification of TIMP-3 residues R20, K22, K45, R48, G49, F50, K76, Y77, R84, R100, K123, K125 and R163 as responsible of recognition of methacrylated and acrylated derivatives is in agreement with previously reported in-silico predictions with a highly sulfated HA tetrasaccharide [57]. In these studies, HDX experiments showed TIMP-3 residues Q155 and W171 clearly participating in recognition. Interestingly, here we only observed these two residues involved in HA_c_ binding. Furthermore, the per-residue energy decomposition analysis indicated that sHA_c_ derivatives recognize a higher number of TIMP-3 residues than HA_c_ (Table 2). Thus, the experimentally observed reduction in the sustained release of TIMP-3 from hydrogels containing sulfated GAG, in comparison to unsulfated GAG, can be correlated with stronger interactions of TIMP-3 with sHA_c_ than with HA_c_ derivatives. Utilizing sHA_c_ as polysaccharide mimicking native GAGs as non-animal-derived alternative allowed to tune the TIMP-3 release profiles of GelMA hydrogels. Likewise, HA-based hydrogels containing dextran sulfate have previously been explored for therapeutic delivery of TIMP-3 after myocardial infarction [19]. GelMA-Hep and starPEG-Hep gels were reported to show a prolonged interleukin-4 release [34,87]. In addition, starPEG-based hydrogels functionalized with sHA tetrasaccharides or polymeric Hep were reported to control the release of positively charged SDF-1 based on their charge properties [88].

The bioactivity of TIMP-3 was preserved especially by GelMA/sHA_c_ gels, as evidenced by its ability to effectively inhibit MMP-9. This aligns with the known role of GAGs in stabilizing bioactive mediator proteins within the ECM [89]. Given the reported short half-life of wild-type TIMP-3, which is less than 4 h [90], the sustained release of TIMP-3 from hydrogels is anticipated to provide therapeutic advantages during critical phases of tissue remodeling by ensuring prolonged bioavailability and activity. In line with this, GelMA/Hep gels were reported to preserve the bioactivity of IL-4 [34]. TIMP-3 released from hydrogels potently inhibited MMP activity by 40 – 85 % over the course of 14 days. In addition, soluble HA_c_ and sHA_c_ and hydrogel extracts did not alter the TIMP-3 induced protease inhibition in a fibroblast-based model. Mechanistically, our structure-based in-silico analysis demonstrated that sHA_c_ recognition sites on TIMP-3 do not overlap with its protease binding site. Likewise, GAG/TIMP-3 complexes were shown to maintain their inhibitory action against MMP-1 and MMP-2 [36,57]. Thus, we expect TIMP-3 to effectively inhibit MMPs regardless of whether it is in a soluble form or bound to sHA_c_.

Since TIMP-3 also modulates angiogenesis through competing with VEGF-A for VEGFR-2 binding, the signaling receptor of VEGF-A that initiates the signaling cascade stimulating angiogenesis [91], its potential anti-angiogenic effects must be considered when used as a therapeutic protein. Soluble TIMP-3 exerted its expected anti-angiogenic activity by inhibiting VEGF-A signaling and endothelial network formation. Notably, sHA_c_, but not HA_c_, neutralized this effect, restoring VEGF-A bioactivity, tube formation, and MVF sprouting. This aligns with reported data showing that sHA can block TIMP-3 binding to VEGFR-2 mainly through electrostatic interactions [92]. Structure-based detailed analysis points to overlapping TIMP-3 interaction sites for sHA_c_ and VEGFR-2, providing a potential mechanistic basis for the loss of anti-angiogenic activity. Thus, the data suggests that sHA_c_ shifts TIMP-3 from an anti-angiogenic to a predominantly protease-inhibitory role, which might be beneficial for wound repair.

No cytotoxic effects of hydrogel extracts were detected in endothelial cells, smooth muscle cells, or fibroblasts, in line with the reported biocompatibility of GelMA, GAGs like HA and sHA, and LAP-based photopolymerization systems [22,29,93]. GelMA/HA_c_ extracts promoted M1 polarization, whereas GelMA and GelMA/sHA_c_ did not, and all formulations reduced M2 differentiation. This is consistent with reports that macrophage responses are highly sensitive to HA modification, with low-molecular-weight HA enhancing inflammatory signaling and highly sulfated HA attenuating it [31,94]. Neither HA_c_ nor sHA_c_ showed anti-factor Xa activity, confirming that sHA_c_ with a low sulfation degree do not introduce heparin-like anticoagulant effects [95].

The M1-promoting effect of GelMA/HA_c_ warranted its exclusion from in-vivo testing. GelMA itself is described as low immunogenic material [96]. However, Zhuang et al. demonstrated that higher intrinsic stiffness of GelMA hydrogels (29 kPa vs. 2 kPa) can promote a more pro-inflammatory M1 macrophage phenotype and modulates the in-vivo inflammatory response [97]. To determine whether the in-vitro immunomodulatory effects translated to an in-vivo setting, we analyzed the host response to TIMP-3-loaded GelMA and GelMA/sHA_c_ hydrogels following subcutaneous implantation. GelMA-based materials have previously been reported to exhibit good biocompatibility in-vivo [98]. The reduced abundance of MPO^+^ neutrophils and CD68^+^ macrophages in the peri-implant granulation tissue of GelMA/sHA_c_ hydrogels compared with GelMA controls indicates that sHA_c_ modification in combination with TIMP-3 loading is associated with attenuated early inflammatory responses, a hallmark of improved biocompatibility. Concomitantly, the higher number of CD31^+^ microvessels suggests enhanced vascular-associated tissue responses around GelMA/sHA_c_ hydrogels. Although TIMP-3 is generally described as anti-angiogenic, context- and concentration-dependent pro-angiogenic effects have been reported [99]. Based on our mechanistic in-silico and in-vitro data, binding of TIMP-3 to sHA_c_ is expected to preserve its protease-inhibitory activity while potentially modulating interactions with receptors such as VEGFR-2, which is consistent with the vascular changes observed in-vivo. The increased Sirius red staining intensity indicates altered collagen-rich matrix characteristics within the granulation tissue in the GelMA/sHA_c_ group compared to the GelMA group. However, this readout cannot distinguish between enhanced matrix deposition and reduced matrix degradation. Given the established role of TIMP-3 as an inhibitor of matrix-degrading proteases, both processes may contribute to the observed histological differences. Overall, these findings indicate that sHA_c_ functionalization of GelMA shifts the peri-implant tissue response toward a more favorable healing environment, characterized by reduced inflammatory cell presence and enhanced vascular- and matrix-associated signatures. Likewise, collagen scaffolds with covalently linked highly sulfated sHA have been shown to modulate inflammatory responses in mice [77]. However, the high degree of sulfation used in those systems is associated with broad, charge-driven protein binding and reported anticoagulant side effects. The lower sulfation degree of sHA_c_ used in the present study does not enter this high-charge interaction regime.

To evaluate the impact of TIMP-3 delivery on matrix degradation, collagen-based artificial ECMs were employed as an in-vitro model. Notably, enzymatic ECM degradation was significantly reduced by TIMP-3 released from GelMA/HA_c_ and GelMA/sHA_c_ hydrogels, even after 7 days. These findings indicate that functionalizing GelMA with HA_c_ and sHA_c_ enhances the sustained release of bioactive TIMP-3, consistent with results from the MMP-9 enzymatic assay. Likewise, GAGs within the ECM are known to functionally protect bioactive mediator proteins [26].

To assess the protease-mediated ECM breakdown ex-vivo, dentin slices were used for monitoring the ability of hydrogel-released TIMP-3 to inhibit protease activity. Our approach aligning with the 3R principle provided a controlled, animal-free platform enabling controlled investigations of therapeutic interventions in biomedical research. Demineralized dentin with its collagen-rich composition and endogenous MMP activity mimics the ECM degradation observed under pathophysiological conditions [74]. Here, the dentin-located inactive proMMPs can be activated by endogenous proteases, reactive oxygen species, and factors such as caries, restorative dental procedures and exogenous agents [41]. Reactivated MMPs like MMP-2, MMP-3, MMP-8, MMP-9 and MMP-20 as well as cathepsins within the dentin play a key role in ECM degradation by cleaving collagen fibrils, even without bacterial involvement, adversely affecting the durability of resin-dentin restorations and increasing susceptibility to secondary caries [100]. The enzymatic activity of dentin after demineralization was in a comparable range as reported previously [74]. TIMP-3 released within 24 h effectively reduced proteolytic activity within the native dentin matrix, irrespective of the hydrogel type evaluated. However, only TIMP-3 delivered via sHA_c_-functionalized hydrogels achieved a significant reduction both in enzymatic activity within the native ECM and collagen degradation after 7 days. This underscores the advantageous role of sHA_c_-functionalization in enhancing the sustained delivery and efficacy of TIMP-3. Incorporating MMP inhibitors into dental adhesives has been suggested as a promising approach to prolong restoration durability [14]. The failure of dental restorations, driven by the degradation of the dentin-resin hybrid layer due to MMP activity, could be mitigated by incorporating MMP inhibitors into dental adhesives or introducing protein-drug conjugates and polymer-based nanoformulations into the exposed matrix to enable sustained release and enzyme inhibition.

Moreover, this study demonstrated that localized delivery of TIMP-3 via GelMA/sHA_c_ hydrogels effectively attenuated ECM degradation in a human ex-vivo skin model. Our data showed that incorporating TIMP-3 into the GelMA/sHA_c_ hydrogel preserved dermal ECM structure more effectively than the hydrogel alone. This likely reflects two synergistic effects: (i) local inhibition of proteases that drive pathological collagen loss, and (ii) stabilization/retention of TIMP-3 at the wound site. These results align with previous findings showing that TIMP-3 inhibits a broad range of MMPs and ADAMTS proteases involved in pathological ECM remodeling in chronic wounds [39]. An imbalanced MMP/TIMP ratio is a hallmark of impaired wound healing [101], and localized TIMP-3 delivery may help restore this balance. Our findings suggest that integrating TIMP-3 into bioactive GelMA-hydrogels offers a promising strategy to preserve ECM integrity in protease-rich wound environments.

Collectively, these findings establish GelMA/sHA_c_ hydrogels as a mechanistically rational platform, in which selectively sulfated sHA_c_ provides tunable affinity sites that decouple TIMP-3-mediated protease inhibition from its anti-angiogenic signaling while enabling sustained release of bioactive inhibitor. In contrast to previous GAG-based delivery systems, our work directly links molecular-level GAG/TIMP-3 recognition with macroscopic release behavior and preservation of ECM integrity in human ex-vivo tissues, thereby outlining a general design principle for ECM-anchored MMP inhibitor delivery using sHA_c_ as a structurally defined, non-animal-derived GAG derivative that avoids the biological variability and anticoagulant risks associated with Hep-based systems.

We acknowledge the potential limitations of this study. While the TIMP-3 release studies were performed over a course of 28 days at body temperature, the simplified buffer composition cannot reflect the complex environment in-vivo. However, since GelMA itself inherently contains MMP cleavable regions, we assume that especially MMP-rich environments would further enhance the TIMP-3 release, which might foster an on-demand local delivery of the MMP inhibitor from the hydrogels. Native GAGs are known for their complexity that is crucial to orchestrate their diverse biological functions. GAG immobilization on biomaterials for medical applications always bears the risk of altering the biological response compared to the soluble GAG form making the replication of GAG complexity into engineered biomaterials even more challenging [11]. Moreover, chemically modified GAG derivatives like sHA_c_ are limited in their ability to replicate the multifaceted biological functions of native GAGs. However, since their structure determines their functions, sHA_c_ derived from sustainable sources with well-defined structures are highly advantageous for potential clinical translation. These materials enable precise control over critical parameters influencing GAG functionality, such as molecular weight and substitution patterns, thereby enhancing their therapeutic potential.

Finally, the assays utilized to investigate the effects of TIMP-3-loaded GelMA/sHA_c_ hydrogels cannot distinguish between inhibitory effects of TIMP-3 and TIMP-3/sHA_c_ complexes released from the hydrogels. However, since the bioactivity of sustainably released TIMP-3 from GelMA/sHA_c_ hydrogels was validated in-vitro and ex-vivo, the evidence strongly suggests the ability of this system to decrease the protease activity in-vivo. Adopting in-vitro and ex-vivo models for biomaterial testing will continue to play a vital role in aligning with the 3R principle, reducing reliance on animal studies, and accelerating the translational pathway to clinical use. Taken together, our data position TIMP-3 loaded GelMA/sHA_c_ hydrogels as locally applicable depots for protease-rich microenvironments, such as demineralized dentin and inflamed skin wounds, where excessive MMP activity accelerates ECM breakdown. By combining defined, non-animal-derived sHA_c_ with an injectable, photocrosslinkable GelMA network and sustained delivery of bioactive TIMP-3, this platform provides a rational basis for future translational studies targeting collagen preservation in dental restorations and chronic or acute dermal wounds.

## 5 Conclusion

In summary, our findings highlight the potential of biomimetic sHA_c_-functionalized hydrogels for localized delivery of therapeutic agents like TIMP-3 to attenuate excessive MMP activity. This study establishes a proof-of-concept for designing GelMA/sHA_c_ hydrogels capable of controlled and sustained release of bioactive MMP inhibitors, minimizing off-target effects and exhibiting favorable peri-implant tissue signatures in-vivo. These hydrogels could be integrated in advanced fabrication techniques, such as 3D bioprinting in the future. The demonstrated therapeutic efficacy following pathophysiological dentin degradation and protease-rich skin wound treatment in human ex-vivo models underscores the need for future preclinical investigations, as this approach could provide a safe and effective treatment for diseases driven by local imbalances between MMPs and TIMPs.

## CRediT authorship contribution statement

**Fabian Junker:** Data curation, Formal analysis, Investigation, Writing – review & editing. **Stefan Rupf:** Conceptualization, Methodology, Resources, Writing – review & editing. **Paula Marie Schindler:** Data curation, Formal analysis, Investigation, Writing – review & editing. **Cedric Wilden:** Data curation, Formal analysis, Investigation, Writing – original draft. **Mathias Hohl:** Methodology, Resources, Writing – original draft. **Gloria Ruiz-Gómez:** Data curation, Formal analysis, Investigation, Writing – original draft. **M. Teresa Pisabarro:** Conceptualization, Data curation, Methodology, Writing – original draft. **Selina Wrublewsky:** Data curation, Investigation, Methodology, Writing – review & editing. **Caroline Bickelmann:** Investigation, Methodology, Writing – original draft. **Charlotte Berhorst:** Data curation, Formal analysis, Writing – review & editing. **Dalia Alansary:** Data curation, Formal analysis, Investigation, Writing – original draft. **Ben Wieland:** Data curation, Formal analysis, Investigation, Writing – original draft. **Markus Bischoff:** Methodology, Resources, Writing – review & editing. **Poh Soo Lee:** Data curation, Formal analysis, Methodology, Writing – review & editing. **Stephanie Moeller:** Data curation, Formal analysis, Investigation, Methodology, Writing – review & editing. **Albrecht Berg:** Data curation, Formal analysis, Investigation, Methodology, Writing – review & editing. **Tobias A. Dancker:** Data curation, Formal analysis, Investigation, Methodology, Writing – review & editing. **Marcel A. Lauterbach:** Data curation, Formal analysis, Investigation, Methodology, Writing – review & editing. **Bergita Ganse:** Formal analysis, Writing – review & editing. **Leticia Prates Roma:** Formal analysis, Resources, Writing – review & editing. **Therese Steudter:** Data curation, Formal analysis, Methodology, Writing – review & editing. **Wolfgang Metzger:** Data curation, Formal analysis, Methodology, Writing – review & editing. **Thomas Tschernig:** Methodology, Resources, Writing – review & editing. **Emmanuel Ampofo:** Data curation, Formal analysis, Methodology, Resources, Writing – original draft. **Matthias W. Laschke:** Methodology, Resources, Writing – review & editing. **Matthias Hannig:** Conceptualization, Methodology, Resources, Writing – review & editing. **Sandra Rother:** Conceptualization, Data curation, Funding acquisition, Investigation, Methodology, Project administration, Supervision, Writing – original draft.

## Ethics approval and consent to participate

All animal experiments were approved by the competent local authorities (Landesamt für Verbraucherschutz, Abteilung C Lebensmittel und Veterinärwesen, Saarbrücken, Germany) and were conducted in accordance with European legislation on the protection of animals used for scientific purposes (Directive 2010/63/EU) and the NIH Guidelines for the Care and Use of Laboratory Animals (http://oacu.od.nih.gov/regs/index.htm. 8th Edition; 2011). Post-mortem human skin biopsies were obtained from body donors who had given written consent for scientific use during their lifetime. The study was approved by the Ethics Committee of the Medical Association of Saarland (approval no. 162/20). Human blood samples from healthy donors were collected at the Institute of Clinical Hemostaseology and Transfusion Medicine, Saarland University, following approval by the local ethics committee (approval no. 18/23 Alansary). No data from social media platforms were collected or analyzed.

## Declaration of competing interest

The authors declare that they have no known competing financial interests or personal relationships that could have appeared to influence the work reported in this paper.
