# Supplementary material for: Glycosaminoglycan-functionalized hydrogels for sustained delivery of tissue inhibitor of metalloproteinase-3 mediating matrix metalloprotease inhibition and extracellular matrix stabilization
Source: Bioact Mater. 2026 Feb 12;61:172–93. doi: 10.1016/j.bioactmat.2026.02.010 (PMC12924898; doi:10.1016/j.bioactmat.2026.02.010)
Supplement: Multimedia component 1 [file mmc1.docx]

**Supporting Information**

**Glycosaminoglycan-functionalized hydrogels for sustained delivery of tissue inhibitor of metalloproteinase-3 mediating matrix metalloprotease inhibition and extracellular matrix stabilization**

**
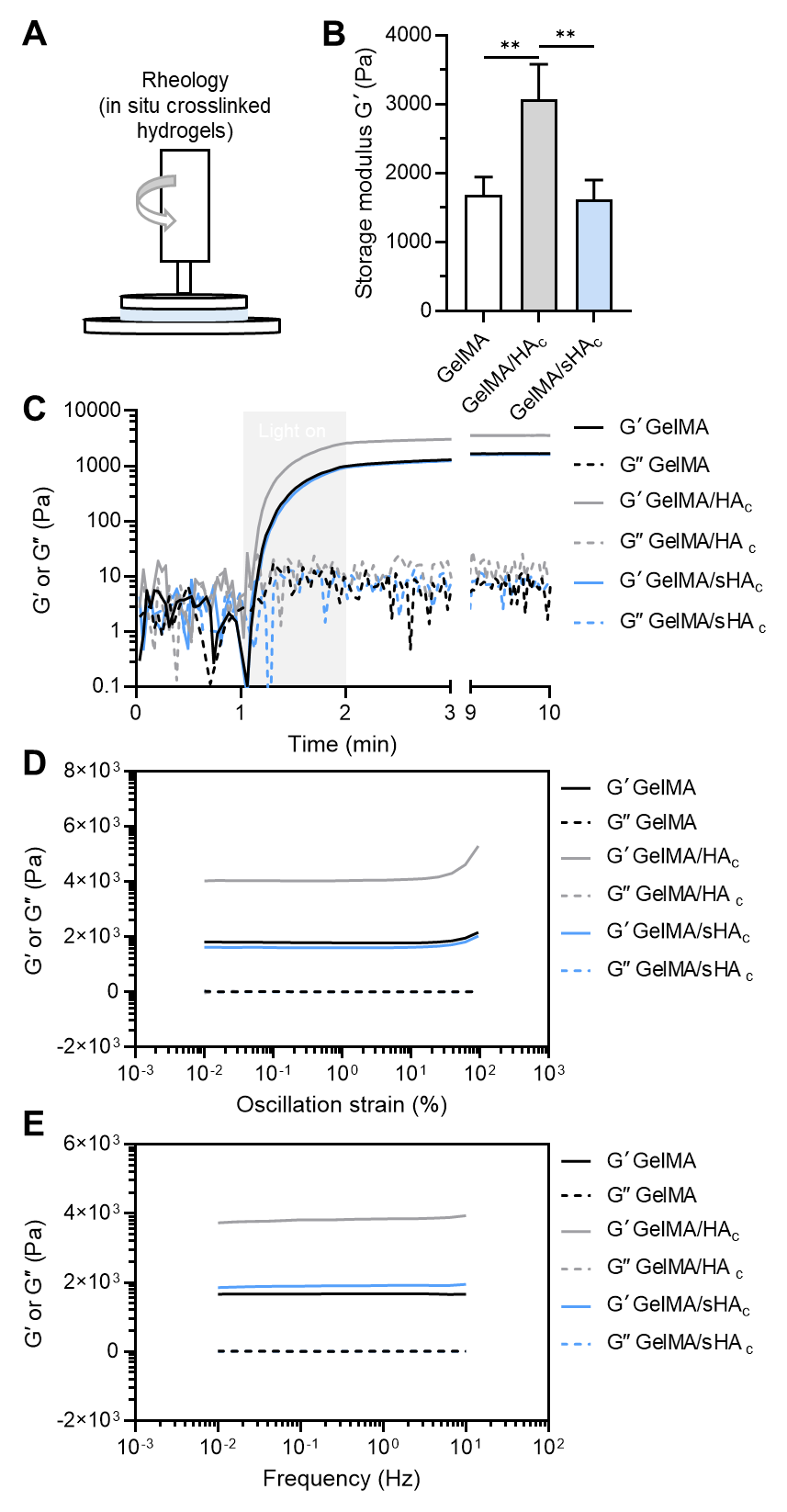
**

**SI Fig. 1 Mechanical characterization of in situ crosslinked hydrogels.** (A) Schematic representation of rheological measurements. The polymer solutions of GelMA, GelMA/HA_c_, and GelMA/sHA_c_ were in situ crosslinked by UV irradiation (60 s) between the rheology plates. (B) Storage modulus (G′) values determined after 10 minutes of crosslinking. (C) Representative oscillatory time sweep curves of in situ crosslinked hydrogels (UV light on, gray area). Illumination started at t = 1 minute. (D) Stress-strain relationships obtained from oscillatory amplitude sweep measurements. (E) Representative oscillatory frequency sweep curves (0.01-10 Hz) of GelMA, GelMA/HA_c_, and GelMA/sHA_c_ hydrogels. One-way ANOVA (**p < 0.01).

**SI Table 1 Comparison of gel point t_gel_, swelling, rheological and compressive data for GelMA-based hydrogels.**

| Hydrogel | GelMA | GelMA/HA_c_ | GelMA/sHA_c_ |
| --- | --- | --- | --- |
| t_gel_ (s) | 17.0 ± 2.5 | 23.6 ± 1.8 | 10.6 ± 1.5 |
| Equilibrium swelling ratio Q (96 h) | 9.8 ± 1.1 | 7.8 ± 2.5 | 9.6 ± 1.7 |
| Polymer volume fraction φ (≈1/Q) | 0.102 | 0.128 | 0.104 |
| Storage modulus G′ (in situ, kPa) | 1.7 ± 0.3 | 3.1 ± 0.5 | 1.6 ± 0.3 |
| Compressive modulus E (rehydrated, kPa) | 16.4 ± 7.6 | 55.0 ± 30.4 | 95.0 ± 21.8 |

**
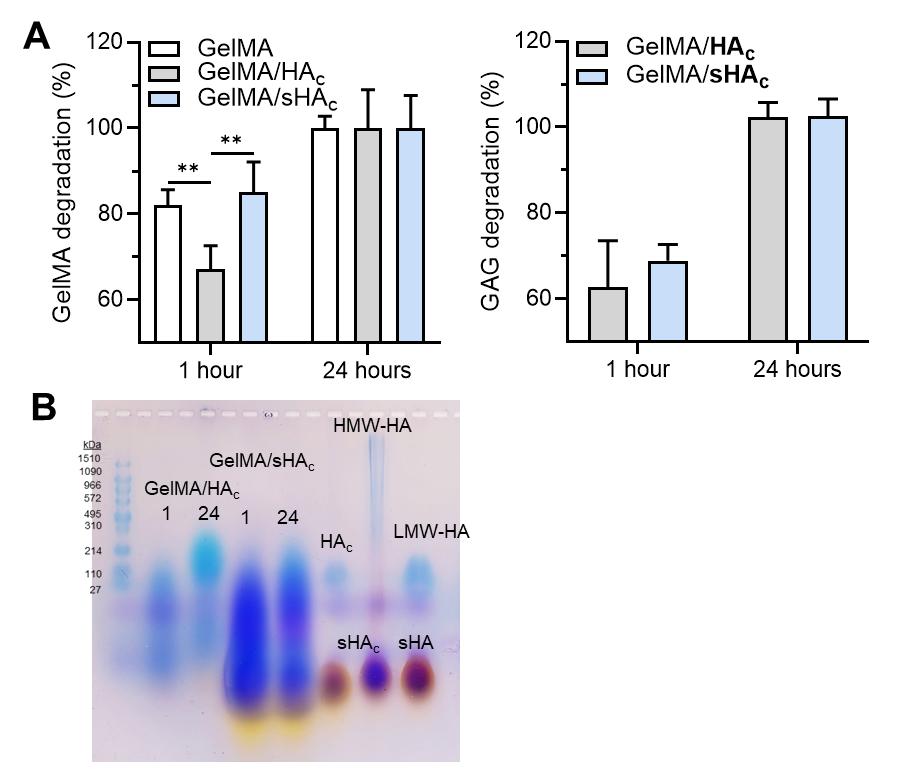
**

**SI Fig. 2 Enzymatic hydrogel degradation.** Hydrogel susceptibility to enzymatic cleavage was evaluated after treatment with a hyaluronidase/collagenase mixture for 1 hour or 24 hours. (A) Degradation products released into the supernatant were quantified using BCA (protein/peptide content), CTAB-based turbidity assay (HA_c_ content), and DMMB (sHA_c_ content). (B) GAGs released from GelMA/HA_c_ and GelMA/sHA_c_ hydrogels were isolated by ethanol precipitation and analyzed by agarose gel electrophoresis followed by Stains-All detection to resolve HA_c_ and sHA_c_ fragments.

**
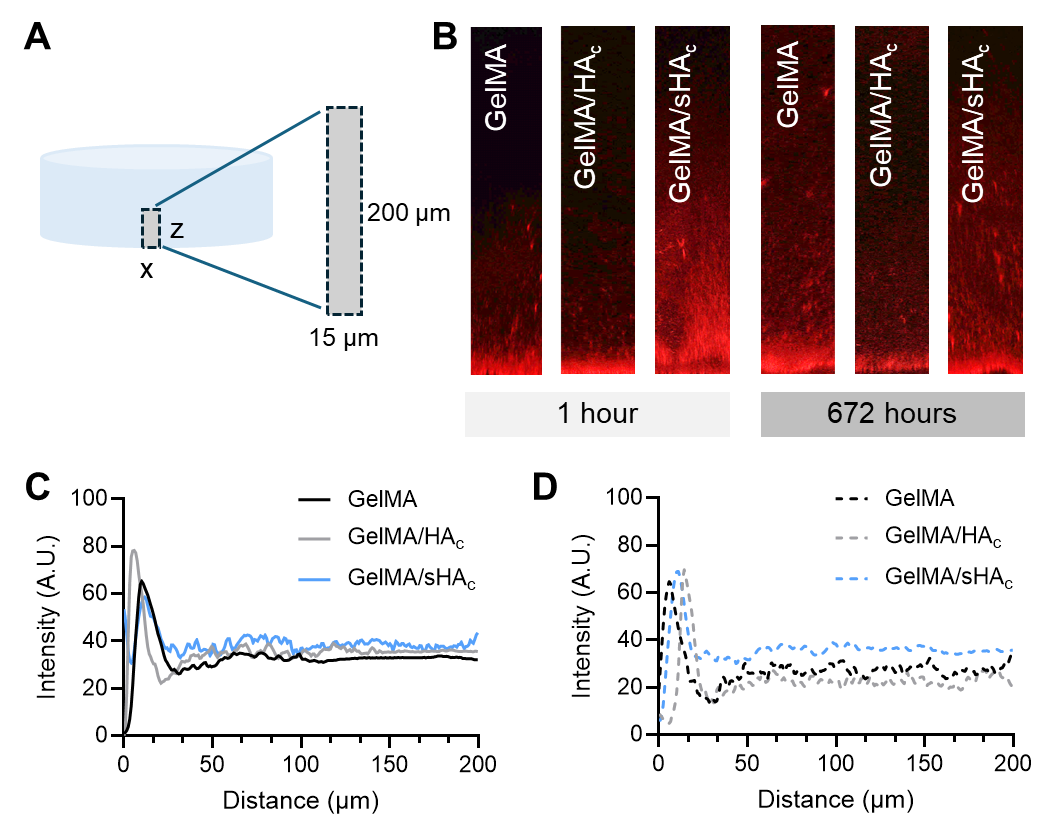
**

**SI Fig. 3 TIMP-3 localization across hydrogel networks.** (A) Schematic illustrating the sample orientation during imaging. (B) Representative confocal images of immunofluorescence-labeled TIMP-3 within the hydrogels at a depth of approximately 200 µm (about 50 % of total hydrogel thickness). (C, D) Corresponding intensity profiles within the hydrogels along the z-axis after 1 hour (C) and after 672 hours (D) analyzed via ImageJ.

**SI Fig. 4 Heatmap illustrating angiogenesis-related protein expression in HUVECs treated with TIMP-3, sHA_c_, or their combination.** Each column represents an angiogenic factor (z-score normalized), and each row a treatment condition.


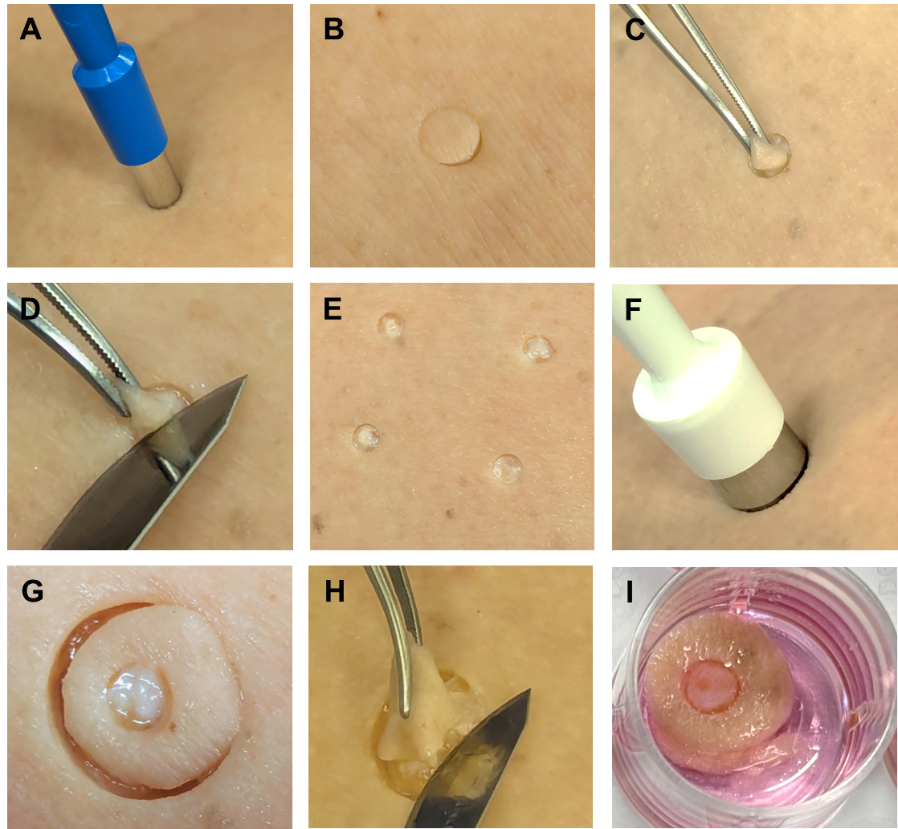


**SI Fig. 5 Sample collection and preparation procedure for the human ex-vivo skin model.** (A, B) 4 mm punch biopsies were created in the skin. (C) The upper skin layer was lifted using forceps. (D) Wounding was induced by removing the upper epidermal layer with a scalpel. (E) Skin samples containing four 4 mm wounds were prepared. (F) A 10 mm biopsy punch was positioned centrally over a 4 mm wound area. (G) The resulting 10 mm biopsy contained a central 4 mm wound. (H) The ex-vivo biopsy was excised from the surrounding tissue using a scalpel. (I) Skin biopsies were subsequently cultured ex-vivo in a 24-well plate.
